# Insect cuticular compounds affect *Conidiobolus coronatus* (Entomopthorales) sporulation and the activity of enzymes involved in fungal infection

**DOI:** 10.1038/s41598-022-17960-z

**Published:** 2022-08-10

**Authors:** Emilia Włóka, Mieczysława Irena Boguś, Anna Katarzyna Wrońska, Mikołaj Drozdowski, Agata Kaczmarek, Justyna Sobich, Marek Gołębiowski

**Affiliations:** 1 Witold Stefański Institute of Parasitology, Polish Academy of Sciences, Twarda, Warsaw, Poland; 2 Laboratory of Analysis of Natural Compounds, Department of Environmental Analysis, Faculty of Chemistry, University of Gdańsk, ul. Wita Stwosza 63, 80-308 Gdańsk, Poland

**Keywords:** Fungal pathogenesis, Enzymes, Infection

## Abstract

Mycoses are a global problem that affects humans and animals. In the present study, the entomopathogenic soil fungus *Conidiobolus coronatus* (Entomophthorales), infecting in tropics also humans, sheep and horses, was cultivated with the addition of insect cuticular compounds (CCs) previously detected in the cuticle of *C. coronatus*—resistant fly species (C10–C30 fatty alcohols, butyl oleate, butyl stearate, glycerol oleate, squalene, tocopherol acetate). Our findings indicate that CCs have diversified and complex effects on the growth and sporulation of *C. coronatus* and its ability to infect the larvae of *Galleria mellonella* (Lepidoptera). The CCs affected protein content and cuticle-degrading enzymes (CDEs) activity in the conidia. Some CCs inhibited fungal growth (0.1% C10), decreased sporulation (C12, C16, C24, C28, C30, butyl stearate, squalene), virulence (C12, C14, butyl oleate, butyl stearate) and protein content (C18). They also reduced conidial CDE activity: elastase (C24, butyl oleate, butyl stearate, squalene, tocopherol acetate), chitobiosidase (C12, C14, C20) and lipase (C12, C18, C26, squalene, tocopherol acetate). Several CCs enhanced sporulation (C14, C18, C22, C26, C30), virulence (C18, C26, squalene), conidial protein content (C16, C24, C30, squalene) and CDE activity: elastase (C10, C16, C18), NAGase (C16, C20), chitobiosidase (C16) and lipase (C10, C14, C16, C20, butyl oleate). Our findings indicate that *C. coronatus* colonies grown on media supplemented with CCs employ various compensation strategies: colonies grown with C16 alcohol demonstrated reduced sporulation but greater conidial protein accumulation and increased elastase, NAGase, chitobiosidase and lipase activity, thus preserving high virulence. Also, colonies supplemented with C18 alcohol demonstrated high virulence and enhanced sporulation and elastase activity but slightly decreased conidial protein content. CCs that inhibit the activity of lipases and proteases show promise in the fight against conidiobolomycosis.

## Introduction

Increases in transplantation surgery procedures, numbers of hospitalized and immunocompromised patients, cases of HIV infection and the need for chemotherapy have resulted in a dramatic rise in incidence of human mycosis over the past few decades. There is a pressing need to develop anti-fungal agents. As infection most commonly occurs by inhalation of conidia, the key to understanding the infection strategies adopted by mycoses, and developing treatments, may well lay in the composition of conidia, particularly the enzymes playing crucial role in the fungal infection progress.

One such fungus is the cosmopolitan soil fungus *Conidiobolus coronatus* (Constantin) belonging to the order Entomophthorales: a typical saprotroph and a facultative human pathogen known to cause mycoses in a broad spectrum of mammals and insects^1–3^. In mammals, entomophthoromycosis manifests as a chronic, inflammatory or granulomatous fungal disease limited to the subcutaneous or submucosal nasal tissue^4^. The first human case of this kind was reported in 1965 in a patient from the West Indies^5^. Most human cases of *C. coronatus* entomophthoromycosis, known as conidiobolomycosis, have been recorded in Africa, South and Central America and south-east Asia^4–7^. Conidiobolomycosis begins in the nasal submucosa and paranasal sinuses, but then spreads to the nasal skin, glabella, cheek, upper lip and pharynx. Lymph node involvement may be observed and in some cases, nasal polyposis and nasal granulomas^6,7^.

In recent years, *C. coronatus* has also gained increasing interest as a potential bio-control agent of insect pests due to the insecticidal properties of its metabolites^8–12^. The present study uses an insect model to study *C. coronatus* infection^13–16^: such models are widely regarded as simple and relatively inexpensive approaches to identifying fungal invasion strategies and developing antifungal agents for preventing human mycoses.

*C. coronatus* acts highly selectively: it kills susceptible insect species rapidly and efficiently, leaving resistant species unhurt^8,9,13^. After adhesion to the cuticle of target insect, the conidium germinates and gives rise to invasive hyphae, which secrete cuticle degrading enzymes (CDEs) such as proteases, chitinases and lipases, these being the main virulence indicators of insecticidal fungi^17^. *C. coronatus* then grows into the host body, reaching the nutrient-rich hemocel and causing the death of the insect in a few days. A study of 43 species of insect from nine systematic groups found that susceptibility to *C. coronatus* infections depends not only on the host species, but also its developmental stage^8,9,13,18^.

Understanding *C. coronatus* resistance in insects is of great value when identifying effective methods of combating conidiobolomycosis in humans. Hence, the fact that the larvae and pupae of scavenger flies demonstrate exceptional resistance to *C. coronatus*, and that fungal spores do not germinate on the cuticle of *Calliphora vicina* larvae^13^, prompted us to analyze the composition of cuticular lipids of these insects^19–26^.

Insects and mammals have evolved different defense systems against fungal invaders, and the course of *C. coronatus* infection differs between the two. However, in both cases, the infection will be initiated by fungal conidia, which overcome the host barrier by enzymatic digestion of the specific components of insect or human tissue, i.e. the proteins, sugars and lipids.

The insect cuticle is the primary adaptive barrier that protects insects against fungal infection, with its outermost surface, the epicuticle, being the key point^27^. The epicuticle is composed of a mixture of lipids, proteins and phenolic compounds that accelerate or inhibit fungal growth; their presence partially determines whether the fungus develops. This layer sits on top of a thicker procuticle consisting mainly of proteins and chitin^27,28^. The epicuticle is itself covered by another layer of saturated and unsaturated hydrocarbons, fatty acids, esters, alcohols, sterols and aldehydes^28,29^.

The cuticular lipid layer of fungus-resistant flies contains a number of cuticle fatty acids, fatty alcohols, esters and sterols, as well as various compounds outside the main group of lipids, such as squalene or tocopherol acetate^19–26^. Several cuticular fatty acids are known to inhibit the growth, sporulation, virulence and toxicity of *C. coronatus*, with these effects depending on the concentration and type of fatty acid^30^.

Fungal invasion of an insect host begins with the digestion of cuticular proteins, chitin and lipids by fungal CDEs (proteases, chitinases and lipases), thus allowing the penetration of fungal hyphae through the three layers of the cuticle. Our previous works show that *C*. *coronatus* cultures produce an enzyme cocktail containing elastase, *N*-acetylglucosoaminidase (NAGase), chitobiosidase and lipase which hydrolyzes the cuticle of *Galleria mellonella*, *Dendrolimus pini, Musca domestica*, *Calliphora vicina*, *C. vomitoria*, *Lucilia sericata*, *Blatta orientalis* and *Blattella germanica *in vitro. The efficiency of digestion was found to depend on the species of insect, resulting in the differential accumulation of amino acids, *N*-glucosamine and free fatty acids as products of protein, chitin and lipid hydrolysis, respectively^13,31–34^.

The aim of this study was to investigate the impact of hitherto poorly-investigated fatty alcohols, esters and the antioxidants squalene and tocopherol acetate (all detected in flies resistant to *C. coronatus* infection), on fungal growth. It also examines their effect on selected determinants of fungal pathogenicity: the ability to infect insects, sporulation efficiency and the CDE profile of the conidia, i.e. the main infective units.

The study also identifies the infection strategies used by the fungus during growth on certain cuticle components by comparing the virulence parameters of *C. coronatus* conidia with their CDE profiles; the findings may guide further research into the treatment of human mycoses. Our research is pioneering: no similar studies have so far evaluated the role of fatty alcohols, fatty esters, squalene and tocopherol acetate in the development of mycoses or fungal pathogenicity. Such substances are already widely used in many branches of industry, including food industries, cosmetics and pharmaceutics.

## Methods

### Fungus

*Conidiobolus coronatus*, isolate number 3747l_−1_, was originally obtained from the collection of Prof. Bałazy (Polish Academy of Sciences, Research Center for Agricultural and Forest Environment, Poznan). The fungus was routinely cultivated on Sabouraud agar medium (SAB) with the addition of homogenized *G. mellonella* larvae at a final concentration of 10% wet weight (SAB-GM) to enhance the sporulation and virulence of fungal colonies. Fungal cultures were maintained in 90 mm Petri dishes at 20 °C under a 12-h L:D photoperiod to stimulate sporulation^35^. Seven-day-old cultures were briefly washed with sterile water in order to harvest conidia. One hundred microliter portions of suspension, each containing approximately 100 conidia, were used for solid media inoculation.

### Addition of tested compounds into the SAB medium

The *C. coronatus* conidia were grown on solid media (SAB) enriched with 16 insect cuticular compounds (CCs) detected in cuticular lipid extracts of *C. vicina*, *C. vomitoria*, *Sarcophaga carnaria* and *M. domestica*^23–26^. The SAB was autoclaved and left to cool to 37 °C. While it was still liquid, the following CCs (Sigma-Aldrich) were added at final concentrations ranging from 0.1 to 0.0001% w/v: C10, C12, C14, C16, C18, C20, C22, C24, C26, C28 and C30 (fatty alcohols), as well as butyl oleate (BO), butyl stearate (BS), glycerol oleate (GO), squalene (S), tocopherol acetate (TA). Most tested compounds remained liquid at room temperature. Long-chain alcohols (C20–C30) were mixed with autoclaved SAB which had been allowed to cool to their melting points (64–87 °C). After thorough mixing, the mixtures were poured onto sterile Petri dishes in a laminar flow hood and sterilized by UV (20 min).

Untreated SAB culture was used as a control. SAB-GM was used as an additional control, as it is the standard medium used in our laboratory for maintaining the high virulence of *C. coronatus* cultures used in testing susceptibility of various insect species. Each experimental variant was performed in three or four independent replications; the controls were performed as seven replications. Cultures were performed for 7 days at the optimum temperature for fungus growth (20 °C).

### Virulence of *C. coronatus* colonies

To determine the ability of *C. coronatus* to infect *G. mellonella*, 40 *G. mellonella* larvae were exposed to each fungus SAB culture supplemented with the CCs at final concentrations of 0.1–0.0001%. All larvae were 5 days old, in their last (VII-th) instar (i.e. at the beginning of the wandering stage, 5 days after last larval molt and 2 to 3 days before pupation), and were maintained and reared in temperature and humidity-controlled chambers (30 °C, 70% r.h.) in constant darkness on an artificial diet^36^. After exposure (20 h), the insects were transferred to clean Petri dishes with food and kept under their growing conditions for 7 days. The larvae at this stage physiologically cease feeding and the food serves them as a safe environment for pupation. The condition of the exposed larvae was monitored daily. The control insects were exposed for 20 h to sterile SAB plates. The exposure of tested insects to a *C. coronatus* colony has been found to be the most efficient method resembling the natural infection process^37^. Virulence was determined based on the percentage of infected larvae. Insect infection was recognized by immobilization of larvae and melanization of the cuticle as black spots. The percentage of dead insects was not taken into account as this work concerns the effect of CCs added to the SAB medium on the levels of cuticle-degrading enzymes (essential for the first phase of infection) in the spores produced by the mycelia grown on such media; in *G. mellonella*, the death of larvae following *C. cor*onatus infection is mainly caused by the toxic metabolites released by the fungus after invasion^8–15,38^.

The conidia of *C. coronatus* were stained with Calcofluor White, which binds to chitin in the fungal cell wall. Conidia washed off the fungus cultures were placed on a µ-Slide 8-well cell culture plate (Ibidi). Two drops of Calcofluor White and two drops of 10% potassium pydroxide (both from Merck) were added to each well. The slide was incubated for one minute at room temperature. To visualize the fungal spores and hyphae on the surface of infected *G. mellonella*, the larvae were placed in the well of a 6-well, flat bottom, sterile plate (Corning). Six drops of Calcofluor White and six drops of 10% potassium pydroxide were added to each well and incubated for one minute at room temperature. The microscope observations and photo documentation were performed using an Axio Vert.A1 fluorescence microscope (Zeiss) with Axio Cam ICc 5 (Zeiss) and ZEN 3.2 lite software with Modul Image Analysis (Zeiss).

### Sporulation efficiency

To estimate the efficiency of *C. coronatus* sporulation, 7-day-old solid cultures were briefly washed with 2 ml sterile water, and the number of harvested conidia was determined under the microscope in a Bürker chamber. Briefly, 50 µl of conidia suspension was thoroughly mixed and taken for counting. For all culture variants, the conidia were counted in three or four independent samples; the controls (SAB and SAB-GM) were counted in seven independent samples. The result was converted into 2 ml and then 1 cm^2^ colony area.

### Protein concentration

Samples with the known number of conidia were sonicated until all conidia were homogenized (inspection under microscope), then the protein contents were measured with the Protein Assay (Bio-Rad). Bovine serum albumin (BSA; Sigma-Aldrich) was used as a standard. The results were based on the total protein content in the entire sample and then calculated as a value per single conidium.

### Enzymatic assays in *C. coronatus* conidia

The sonicated samples of conidia were centrifuged (4 °C, 20 min, 1500 × *g*). Activities of the cuticle degrading enzymes (CDEs) were measured in supernatants as described earlier^31^. The activities of elastase, *N*-acetylglucosaminidase (NAGase), chitobiosidase and lipase, all considered as determinants of fungal virulence, were measured spectrophotometrically and spectrofluorimetrically (BioTek Synergy HT) in 96-well polystyrene plates (Corning) using suitable synthetic substrates (all from Sigma-Aldrich) made up to a final volume of 200 μl with reaction buffer (Tris–HCl pH 7.0, 8.0 or 10.0—depending on the substrate used). All supernatant samples were thoroughly mixed for one minute before adding to the appropriate substrate solutions.

Elastase activity was measured in 2 μl of supernatant samples mixed with 0.5 mM N-Succinyl-Ala-Ala-Pro-Leu-p-Nitroanilide in a 100 mM Tris–HCl buffer containing 20 mM CaCl_2_ (pH 8.0). Absorbance was read at 410 nm. NAGase activity was measured using 5 μl of supernatant samples incubated with 0.3 mM 4-Nitrophenyl-*N*-acetyl-β-d-glucosaminide in a 10 mM Tris–HCl buffer (pH 7.0). Absorbance was read at 405 nm. Chitobiosidase activity was measured in 6 μl of supernatant samples added to 0.003 mM 4-Methylumbelliferyl β-d-N–N′-diacetylchitobioside in a 50 mM Tris–HCl buffer (pH 7.0). Fluorescence was read at Ex = 340 nm, Em = 450 nm. Lipase activity was measured using 10 μl of supernatant samples incubated with 0.01 mM 4-Methylumbelliferyl oleate in a 50 mM Tris–HCl buffer (pH 10.0). Fluorescence was read at Ex = 360 nm and Em = 450 nm. All reaction mixtures were incubated at 30 °C. The assays were prepared in three or four independent replicates.

CDEs activities were presented as: (a) total activity of the whole sample calculated per ng of conidial protein present in the sample, (b) activity calculated per ng of proteins present in single conidium, (c) activity calculated per single conidium.

### Statistics

Statistical analysis was performed using STATISTICA 6.1 software (StatSoft Polska). Statistical relationships were evaluated using the two-way ANOVA, with Dunnett’s test for post hoc analysis. The Kolmogorov–Smirnov (K–S) test was used to check normality. Principal component analysis (PCA) was used to visualize differences in the effects of various CCs on the main factors determining the effectiveness of fungal infection.The PCA test was performed using Past 4.05 software^39^. Differences in virulence were estimated using a generalized linear model with binary distributions and logit link function in Statistica 6.1. Concentrations were set as a continuous predictor, compounds were set as a qualitative predictor, and infection response (infected/uninfected larvae) was set as a binary dependent variable. The chi-square test was used for post hoc comparison.

## Results

### Sporulation of *C. coronatus*

**Figure 1:**
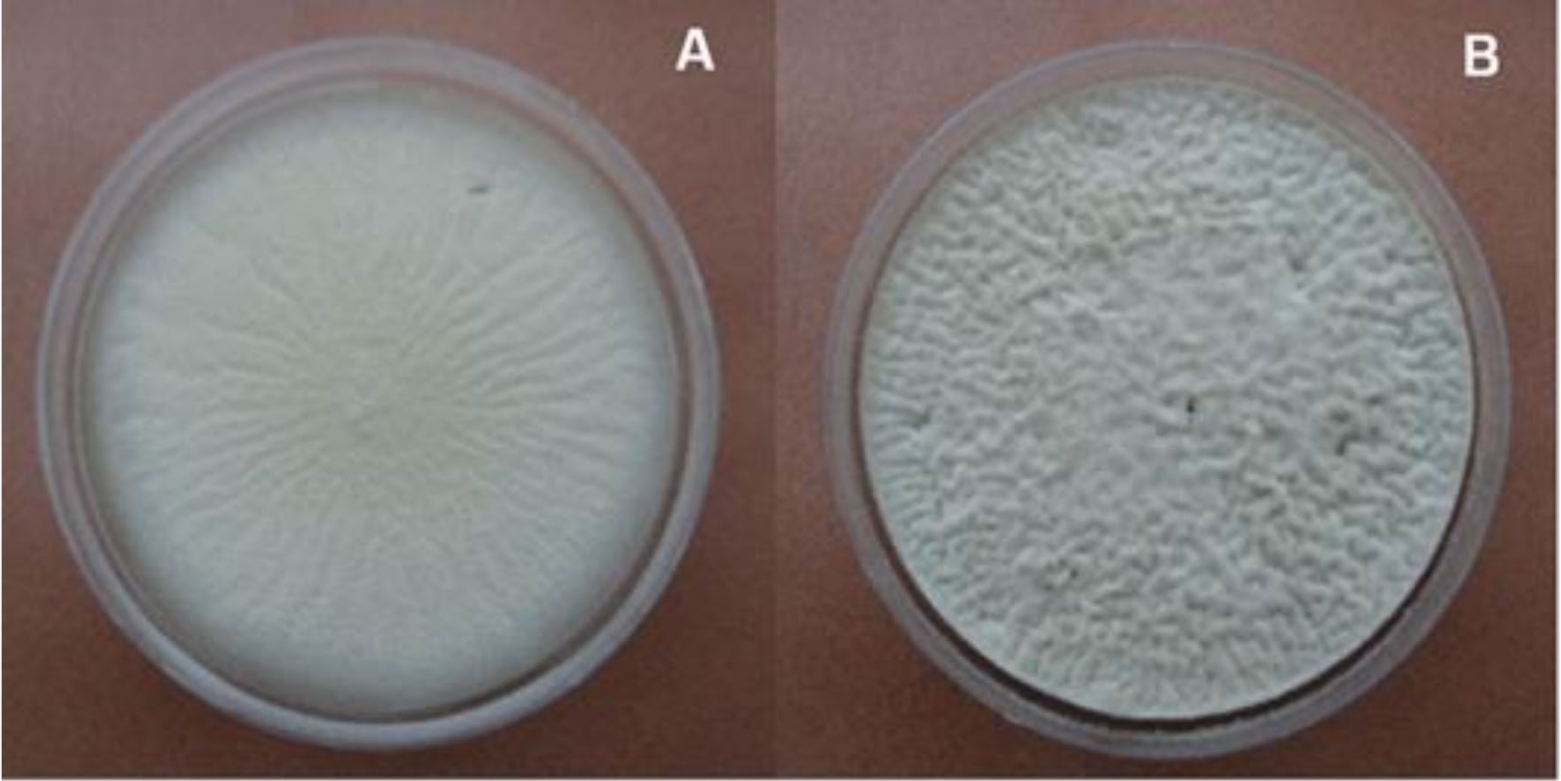
*Conidiobolus coronatus* cultures on SAB (**A**) and SAB-GM (**B**) media.

Cultivation on solid media, as described in Methods, is a standard procedure used to determine the ability of a filamentous fungus to sporulate. Figure 1 presents *C. coronatus* cultures on Sabouraud agar (SAB) (Fig. 1A) and SAB medium enriched with a homogenate of *G. mellonella* (SAB-GM) (Fig. 1B). The mycelia growing on SAB-GM had a more undulating surface than those on the SAB alone. In contrast, supplementation of SAB medium with all tested cuticular compounds (CCs) had no influence on the mycelium appearance. No differences were found in the appearance and size of spores produced by mycelia grown on control SAB and SAB-GM media and on media supplemented with the CCs.

**Figure 2:**
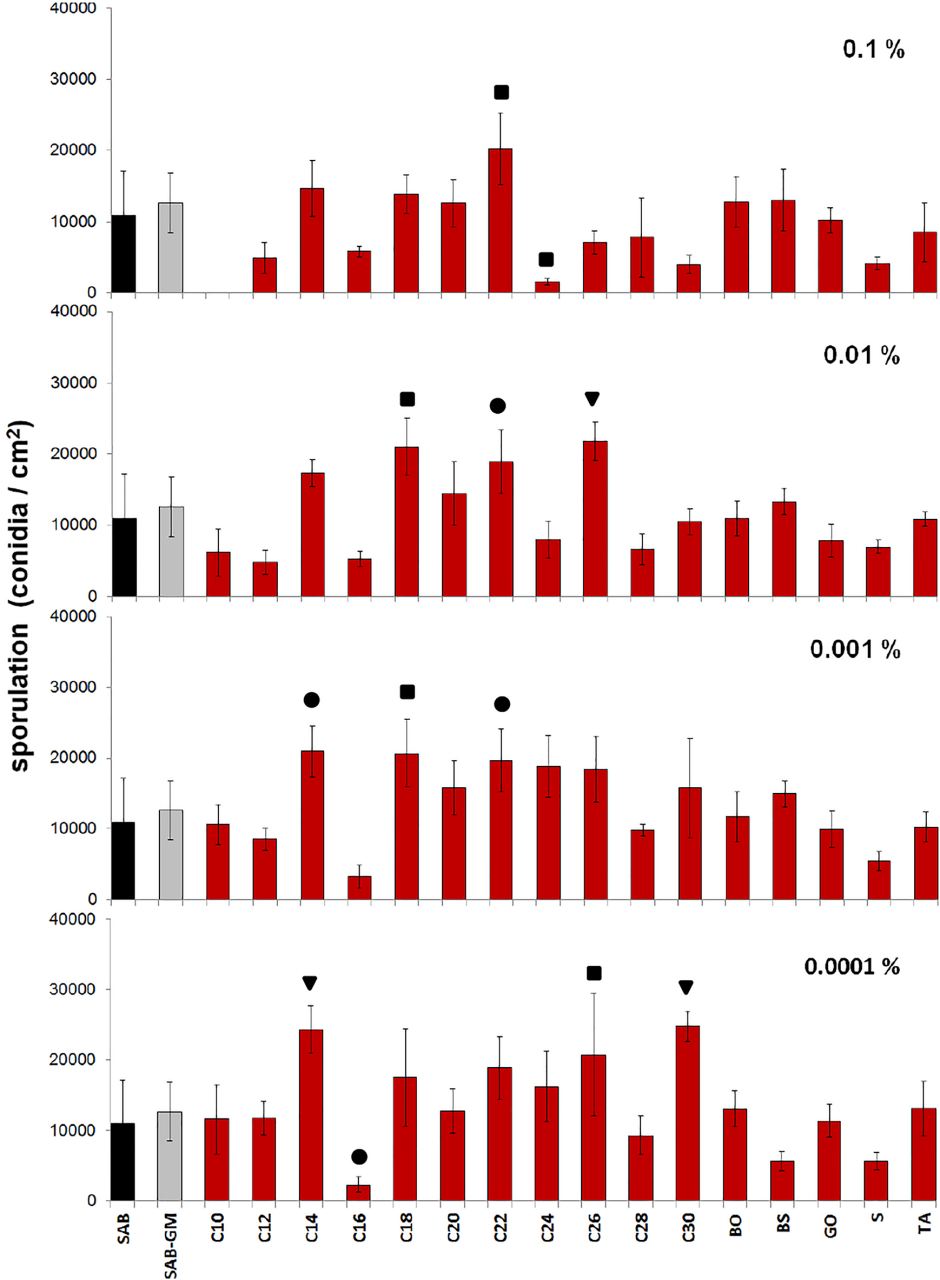
Effect of cuticular compounds (CCs) on the sporulation of *C. coronatus*. Bars indicate means ± SD. *C10–C30* fatty alcohols, *BO* butyl oleate, *BS* butyl stearate, *GO* glycerol oleate, *S* squalene, *TA* tocopherol acetate. Control: amount of conidia produced by *C. coronatus* on SAB (10,900 ± 6282 conidia/cm^2^, N = 7), additional control: amount of conidia produced by *C. coronatus* on SAB-GM (11,880 ± 4345 conidia/cm^2^, N = 8). Statistical analysis: two-way ANOVA, Dunnett *posthoc* test. Data differing significantly from SAB: ● *p* < 0.05; **■** *p* < 0.001;▼ *p* < 0.0001.

Sporulation appeared to be influenced by the type of CC used to supplement the SAB medium and their applied concentrations (Fig. 2; see also Table 1); however, the differences in sporulation between SAB and SAB-GM were statistically insignificant. The highest conidia production (24,809 ± 2155 conidia/cm^2^) was observed after SAB supplementation with 0.0001% C30 alcohol; this value was about 2.3-fold higher than in the case of fungus cultured on SAB alone (10,900 ± 6282 conidia/cm^2^; *p* < 0.001) and 2.0-fold higher than in SAB-GM cultures (11,880 ± 4345 conidia/cm^2^). In contrast, the addition of 0.1% C10 alcohol completely inhibited fungal growth and sporulation, but further dilutions had no effect on sporulation. Similarly, SAB supplemented with 0.1% C24 alcohol significantly inhibited sporulation (1609 ± 434 conidia/cm^2^) by 6.8-fold compared with SAB (*p* = 0.008). A low concentration (0.0001%) of C16 also significantly reduced sporulation (2293 ± 1038 conidia/cm^2^; *p* = 0.020).

**Table 1:**
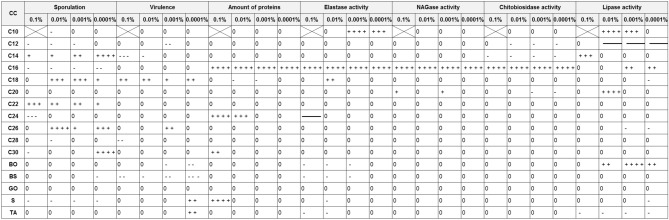
The effect of cuticular compounds (CCs) on sporulation, virulence and cuticle degrading enzymes (CDEs) activity of *C. coronatus* conidia.

*CC* cuticular compound, *C10–C30* fatty alcohols, *BO* butyl oleate, *BS* butyl stearate, *GO* glycerol oleate, *S* squalene, *TA* tocopherol acetate.

+ + + + very strong stimulation (*p* < 0.001); + + + strong stimulation (*p* < 0.01); + + moderate stimulation (*p* < 0.5); + slight stimulation (*p* > 0.05);- - - - very strong inhibition (*p* < 0.0001); - - - strong inhibition (*p* < 0.01); - - moderate inhibition (*p* < 0.05); - slight inhibition (*p* > 0.05); 0 lack of any effect;  lack of growth;  lack of activity.

In contrast, fungus grown on SAB demonstrated enhanced sporulation following supplementation with low concentrations of C14 alcohol (0.001 and 0.0001%; 21,010 ± 3602 and 24,272 ± 3416 conidia/cm^2^; *p* = 0.002 and *p* < 0.001, respectively). Significant stimulation was also observed for the following alcohols: C18 (0.01 and 0.001%; 21,076 ± 3995 and 20,679 ± 4800 conidia/cm^2^; *p* = 0.002 and *p* = 0.004, respectively), C22 (0.1–0.001%; from 20,238 ± 2151 to 18,827 ± 4467 conidia/cm^2^; *p* range: 0.040–0.008, respectively) and C26 (0.01 and 0.0001%; 21,825 ± 2665 and 20,723 ± 8629 conidia/cm^2^; *p* < 0.001 and *p* = 0.003, respectively).

### Virulence of *C. coronatus*

Larval infection was found to occur during 2 days after contact with *C. coronatus* (Supplementary Fig. 1). In the first stage of infection, black melanized spots were observed on the cuticle followed by immobilization of larvae and the cessation of silk spinning and construction of cocoons. Infection results in the death of the insect and its mummification.

**Figure 3:**
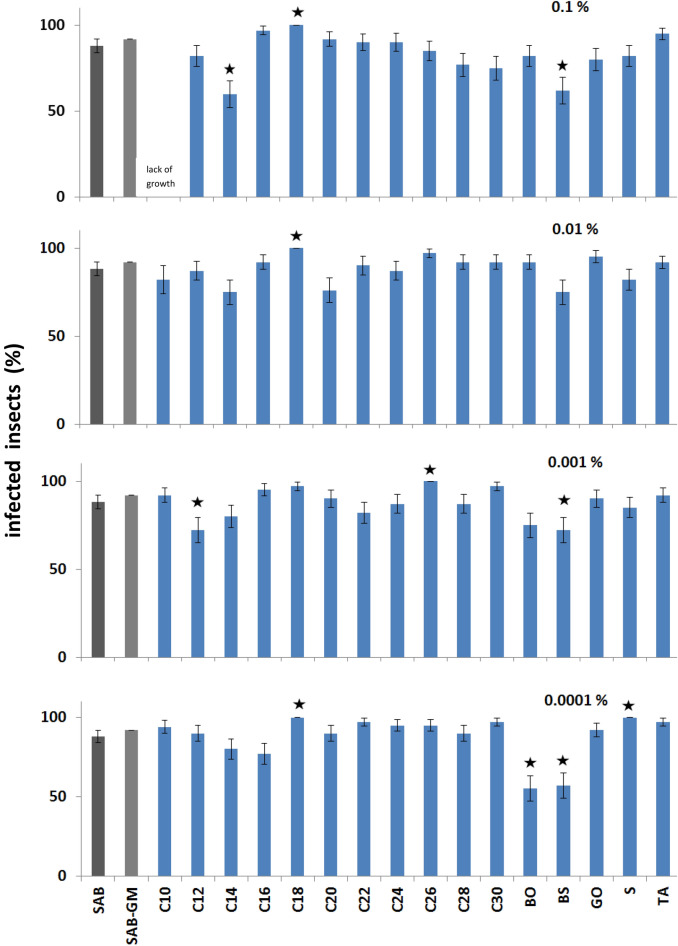
Effect of cuticular compounds (CCs) added to SAB medium on the ability of *C. coronatus* colonies to infect *G. mellonella* larvae. Bars indicate means ± SE (standard error calculated by the logit regression method). Effect of each CC on fungal virulence was tested as described in “Methods” section. Each test was performed on 40 *G. mellonella* larvae. *C10–C30* fatty alcohols, *BO* butyl oleate, *BS* butyl stearate, *GO* glycerol oleate, *S* squalene, *TA* tocopherol acetate. Control: fungal colonies grown on SAB (88 ± 7% of infected larvae, N = 70), additional control: fungal colonies grown on SAB-GM (92 ± 6% of infected larvae, N = 70). Statistical analysis: chi-square test, data differing significantly from SAB *p* < 0.05.

Exposure of *G. mellonella* larvae to fungus grown on SAB and SAB-GM resulted in 88 ± 7% and 92 ± 6% mycosis of the tested populations, respectively. Most of the applied CCs had no significant effect on *C. coronatus* virulence, with few exceptions (Fig. 3; see also Table 1). No significant differences in virulence were observed between the SAB and SAB-GM controls. Of all the tested compounds, only the addition of C18 alcohol elevated the virulence of *C. coronatus*, resulting in 100% mycosis across the whole range of applied concentrations; this increase was statistically insignificant compared to the SAB control only at 0.001% concentration (*p* > 0.05). Significantly elevated virulence was recorded after SAB supplementation with C26 (0.001%; *p* = 0.026) and squalene (S) (0.0001%, *p* = 0.026).

In contrast, the addition of C12 (0.001%), C14 (0.1%), BO (0.0001%), and BS (0.1, 0.001 and 0.0001%) significantly decreased the ability of *C. coronatus* to infect *G. mellonella* larvae (*p* range: 0.032–0.0002).

Concentration-dependent effects in reducing virulence were observed for C14 (Wald test W = 9.5, *p* = 0.002), while opposite effects in terms of increasing virulence were seen after C30 supplementation (W = 10.5, *p* = 0.001). Pair wise comparison for all concentrations (concentration input as continuous predictor) revealed significant differences between BO and C10, C18, C20, C22, C24, C26, C28, C30, GO, S, TA (W range: 4.5–19.9, *p* range: 0.033–*p* < 0.001); BS and C10, C12, C16, C18, C20, C22, C24, C26, C28, GO, S, TA (W range: 8.7–32.2, *p* range: 0.003–0.000). Significant differences were also revealed when comparing C12 with C18, C26, TA (W range: 6.7–11.4, *p* range: 0.009–*p* < 0.001); C14 with C16, C18, C20, C22, C24, C26, C28, C30, GO, S, TA (W range: 6.8–23.3, *p* range: 0.009–*p* < 0.001); C18 with C20, C22, C24, GO, S (W range: 4.7–6.6, *p* range: 0.028–0.009); C28 with TA (W = 4.0, *p* = 0.043).

### Protein content in *C. coronatus* conidia

**Figure 4:**
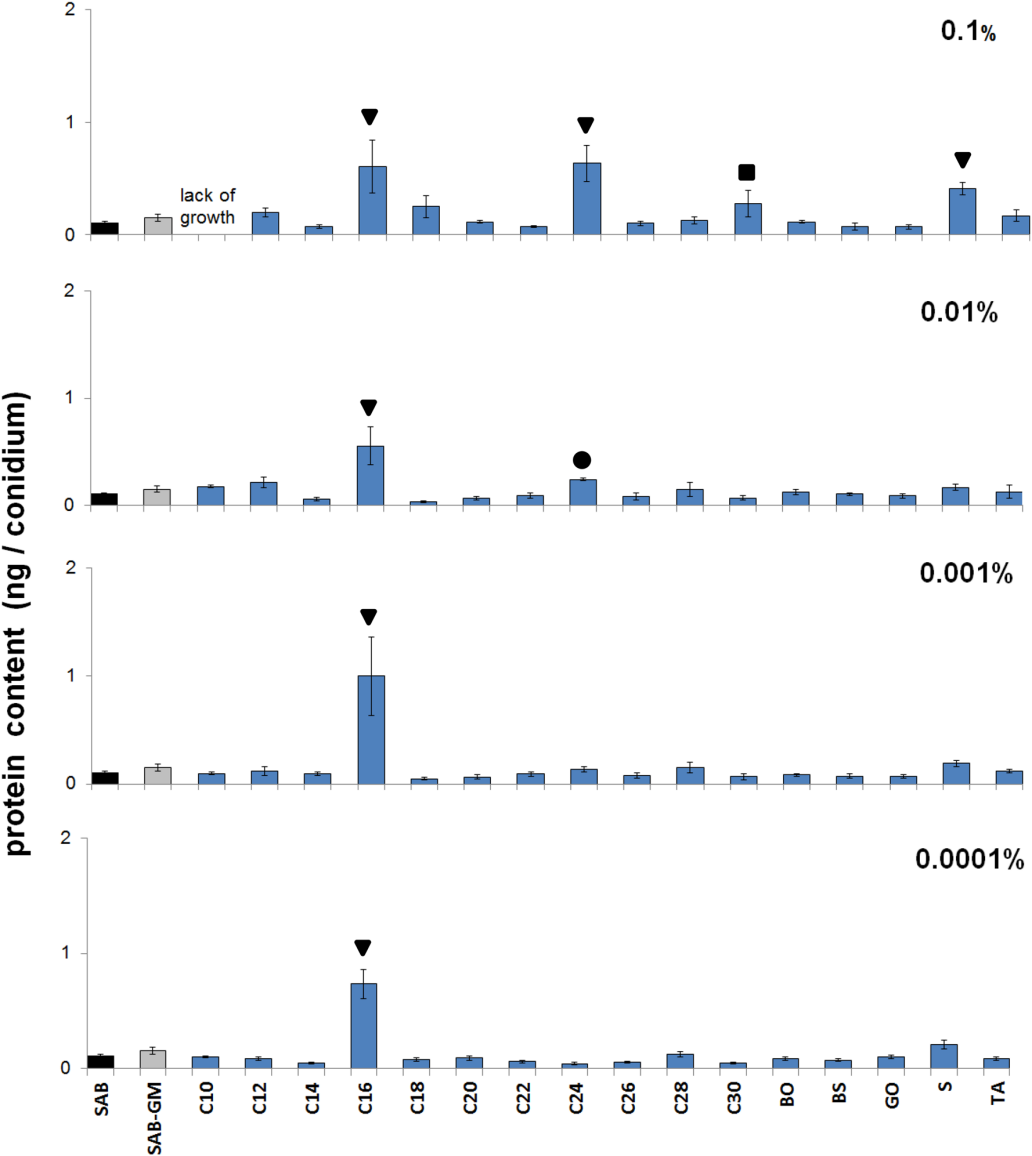
Effect of cuticular compounds (CCs) on protein content in *C. coronatus* conidia*.* Bars indicate means ± SD (when bigger than data points). N = 4 for each concentration of each CC. *C10–C30* fatty alcohols, *BO* butyl oleate, *BS* butyl stearate, *GO* glycerol oleate, *S* squalene, *TA* tocopherol acetate. Control: protein content in conidia produced by fungal colonies grown on SAB (0.11 ± 0.01 ng/conidium, N = 7). Additional control: protein content in conidia produced by fungal colonies grown on SAB-GM (0.15 ± 0.03 ng/conidium, N = 7). Statistical analysis: two-way ANOVA, Dunnett *posthoc* test. Data differing significantly from SAB: ● *p* < 0.05; **■** *p* < 0.001;▼ *p* < 0.0001.

The effect of SAB supplementation with tested CCs on protein content in *C. coronatus* conidia is shown in Fig. 4 and Table 1. The conidia collected from SAB enriched with *G. mellonella* larval homogenate (SAB-GM) displayed 1.4-fold higher protein content compared with SAB alone, however the differences in protein content between conidia from SAB and SAB-GM were statistically insignificant. More precise protein content values in *C. coronatus* conidia are given in Supplementary Table 1.

The addition of C16 alcohol to SAB at all used concentrations (0.1–0.0001%) significantly elevated conidium protein content (in all cases, *p* < 0.001). The remaining CCs caused an increase in protein content when applied at various concentrations: C24 (0.1 and 0.001%; *p* < 0.001 and *p* = 0.020, respectively), C30 (0.1%; *p* = 0.005) and S (0.1%; *p* < 0.001).

No significant decrease in protein content was observed after SAB supplementation with any CC.

### Elastolytic activity in conidia of *C. coronatus*

**Figure 5:**
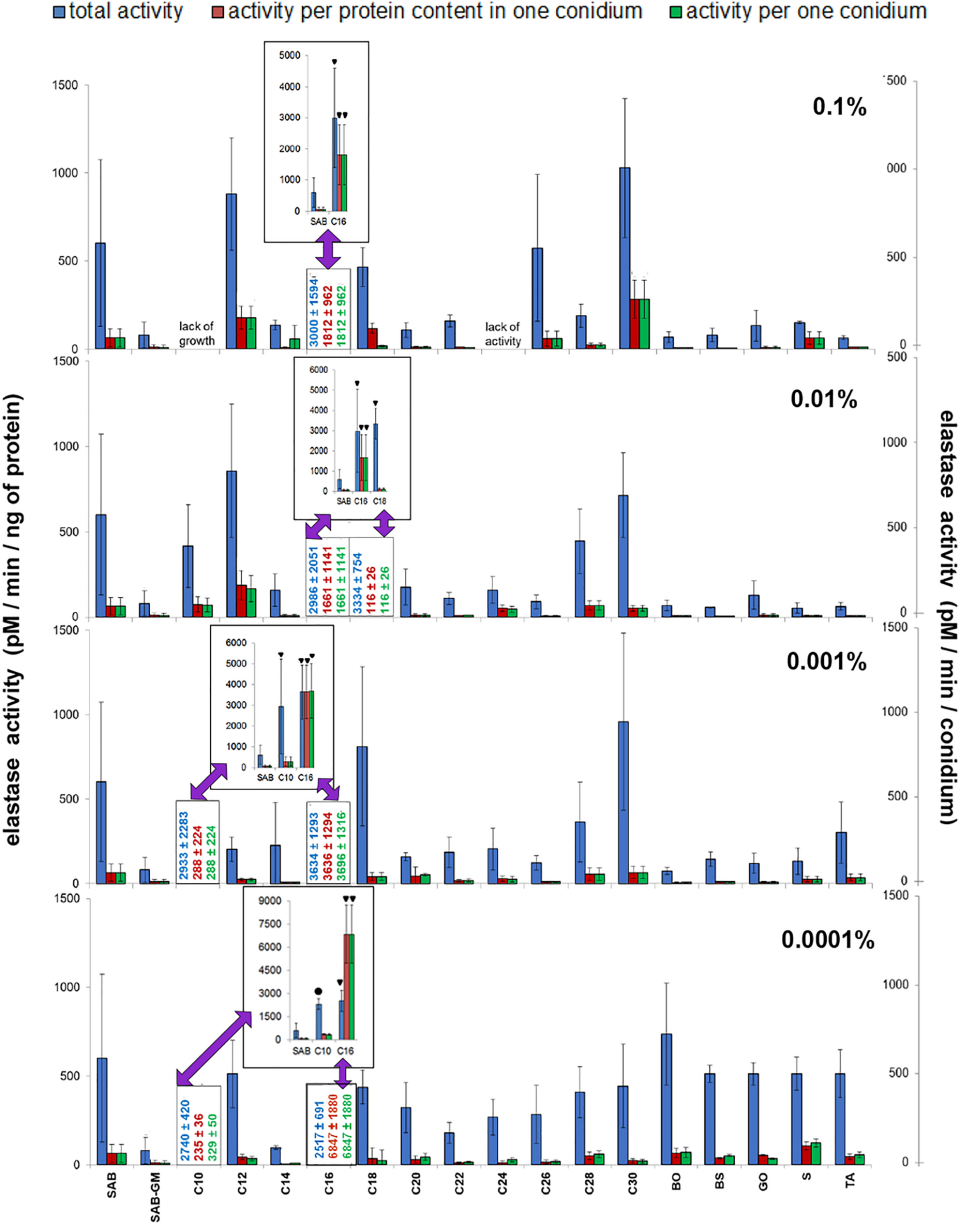
Effect of cuticular compounds (CCs) on elastase activity in conidia homogenates. Bars indicate means ± SD (when larger than data points). *C10–C30* fatty alcohols, *BO* butyl oleate, *BS* butyl stearate, *GO* glycerol oleate, *S* squalene, *TA* tocopherol acetate. Control: total elastolytic activity in conidia from SAB (600.82 ± 472.24 pM/min/ng of protein, N = 7), additional control in conidia from SAB-GM (80.25 ± 73.89 pM/min/ng of protein, N = 7). CC values, which do not fit on Y-axis scale, are given as numerical values, and as bar graphs (insets). Statistical analysis: two-way ANOVA, Dunnett *posthoc* test. Data differing significantly from SAB: ● *p* < 0.05; **■** *p* < 0.001; ▼ *p* < 0.0001.

Elastase plays a key role in the infection process. Only few of the tested CCs affected elastase activity in *C. coronatus* conidia (Fig. 5; see also Table 1). More precise values are given in Supplementary Table 2. Differences in total elastase activity observed between conidia from SAB and SAB-GM cultures (601 ± 472 and 80 ± 74 pM/min/ng of protein, respectively) were statistically irrelevant. In this context, total elastase activity is understood as the elastase activity of all sonicated conidia present in the sample calculated per protein content in the sample.

Interestingly although fungal growth was observed in the presence of 0.1% C24 alcohol, no elastolytic activity was detected. Furthermore, while the addition of 0.1% C10 alcohol inhibited fungal growth, lower concentrations, viz*.* 0.001 and 0.0001%, caused a significant increase of elastase activity (in both cases, *p* < 0.001). For alcohol C10, the greatest elastase activity was measured at a concentration of 0.001%, i.e. a 4.9-fold increase compared with untreated SAB, while for C18, the highest elastase activity was measured at 0.01%: a 5.5-fold increase compared with SAB (*p* < 0.001).

It should be noted that the elastolytic activity measured for 0.001–0.0001% C10, 0.1–0.0001% C16 and 0.01% C18 in comparison with SAB alone, was very high and the data would not fit as bars on the graph. Therefore, numerical values were plotted on the X-axis instead of bars, and the bars are presented in insets of smaller bar graphs: the numerical values of the smaller bar graphs are indicated by arrows (Fig. 5).

C16 increased total elastase activity at all applied concentrations (0.1–0.0001%; in all cases, *p* < 0.001), with the highest enzyme activity occurring at a concentration of 0.001% (sixfold increase compared with SAB control; 3634 ± 1293 pM/min/ng of protein). In contrast, supplementation with 0.01% BO, BS, S and TA slightly (statistically insignificant) decreased elastase activity.

**Figure 6:**
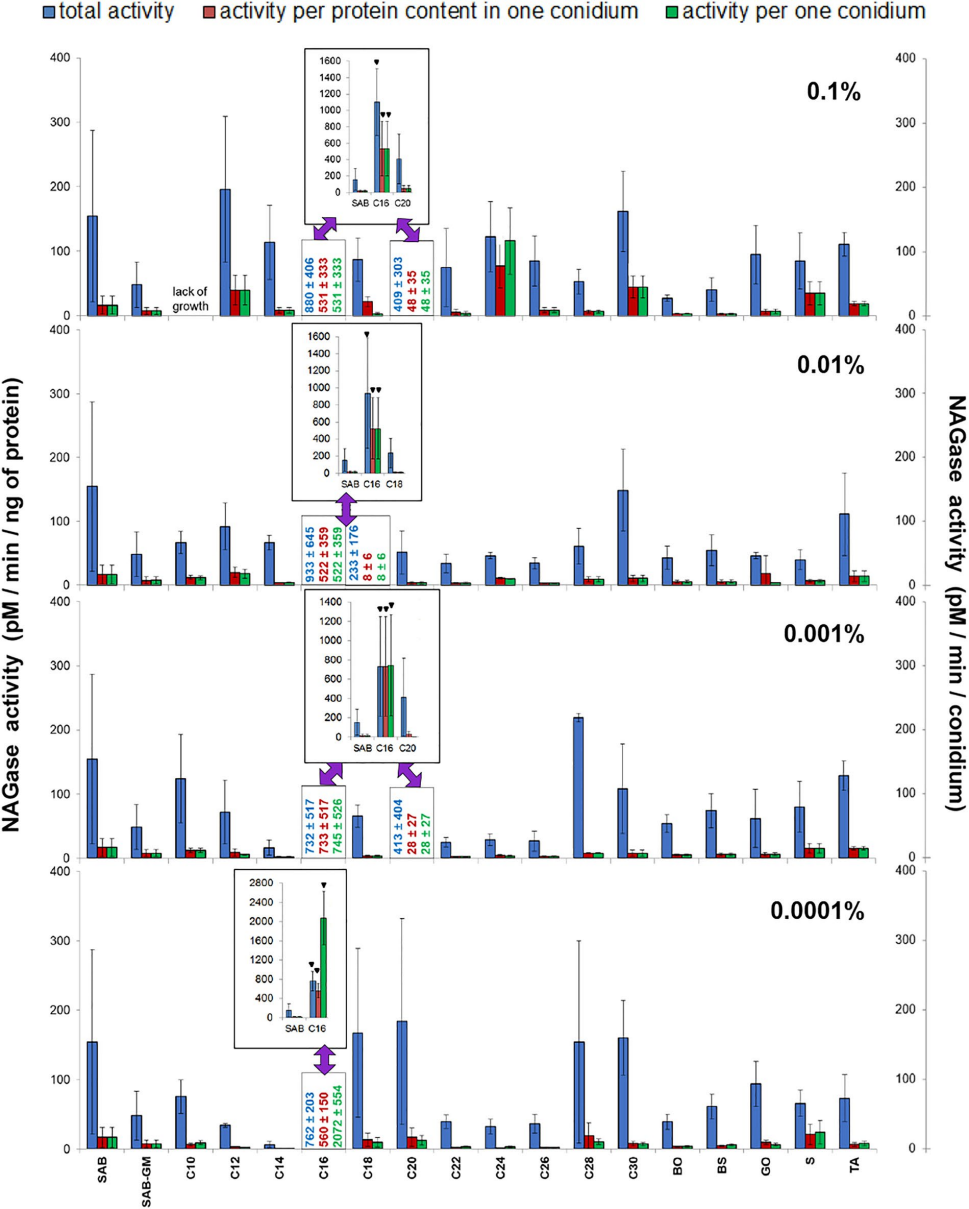
Effect of insects cuticular compounds (CCs) on NAGase activity in conidia homogenates. Bars indicate means ± SD (when larger than data points). *C10–C30* fatty alcohols, *BO* butyl oleate, *BS* butyl stearate, *GO* glycerol oleate, *S* squalene, *TA* tocopherol acetate. Control: total NAGase activity in conidia from SAB (154.46 ± 132.95 pM/min/ng protein, N = 7). Additional control: total NAGase activity in conidia from SAB-GM (48.15 ± 35.09 pM/min/ng protein, N = 7). CC values which do not fit the Y-axis scale are given as numerical values, and are presented in bar graphs (insets). Statistical analysis: two-way ANOVA, Dunnett *posthoc* test. Data differing significantly from SAB: ● *p* < 0.05; **■** *p* < 0.001; ▼ *p* < 0.0001.

**Figure 7:**
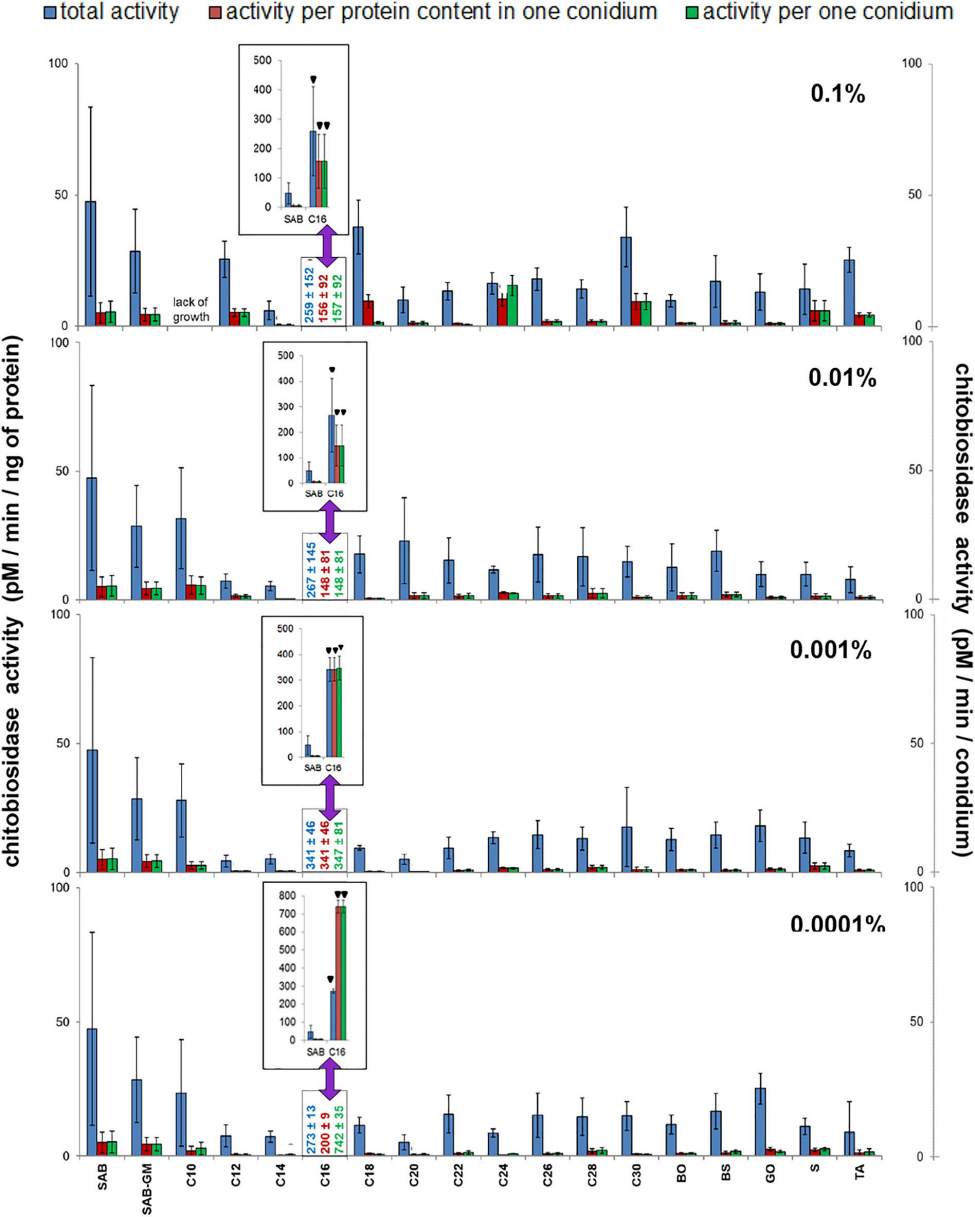
Effect of insect cuticular compounds (CCs) on chitobiosidase activity in conidia homogenates. Bars indicate means ± SD (when larger than data points). *C10–C30* fatty alcohols, *BO* butyl oleate, *BS* butyl stearate, *GO* glycerol oleate, *S* squalene, *TA* tocopherol acetate. Control: total chitobiosidase activity in conidia from SAB (47.36 ± 35.96 pM/min/ng protein, N = 7); Additional control: total chitobiosidase activity in conidia from SAB-GM (28.51 ± 15.91 pM/min/ng protein, N = 7). CC values which do not fit the Y-axis scale are given as numerical values, and are presented in bar graphs (insets). Statistical analysis: two-way ANOVA, Dunnett *posthoc* test. Data differing significantly from SAB: ● *p* < 0.05; **■** *p* < 0.001; ▼ *p* < 0.0001.

**Figure 8:**
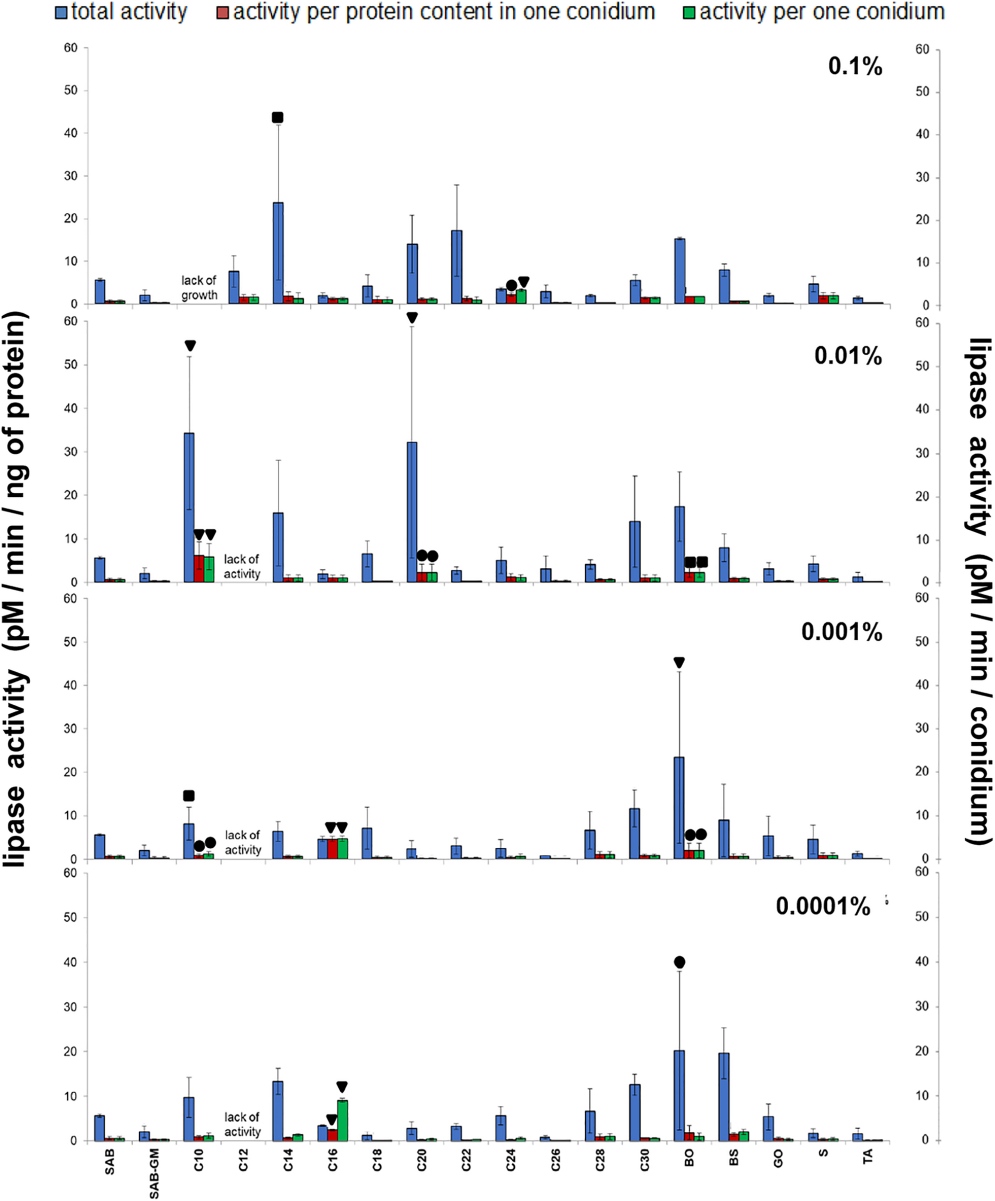
Effect of insect cuticular compounds (CCs) on lipase activity in conidia homogenates. Bars indicate means ± SD (when bigger than data points). *C10–C30* fatty alcohols, *BO* butyl oleate, *BS* butyl stearate, *GO* glycerol oleate, *S* squalene, *TA* tocopherol acetate. Control: total lipolytic activity in conidia from SAB (5.60 ± 3.03 pM/min/ng protein, N = 7). Additional control: total lipolytic activity in conidia from SAB-GM (2.01 ± 1.26 pM/min/ng protein, N = 7). Statistical analysis: two-way ANOVA, Dunnett *posthoc* test. Data differing significantly from SAB: ● *p* < 0.05; **■** *p* < 0.001; ▼ *p* < 0.0001.

It should be pointed out that these results were acquired by measuring the activity of elastase in homogenates made from all fungal conidia collected from mycelia (Fig. 5). However, the fungus produced different numbers of conidia under different culture conditions (Fig. 2) and the conidia had different protein contents (Fig. 4). The general elastolytic activity measured in the conidia was influenced by three variables: the number of conidia produced by the mycelium, the protein content of each conidium, and the proportion of proteins with elastolytic activity in each conidium. To correctly determine the contribution of each of these factors to the overall enzymatic activity, the elastolytic activity is presented in three ways: (a) total activity of the whole sample calculated per ng of conidial protein present in the sample, (b) activity calculated per ng of proteins present in single conidium, and (c) activity calculated per single conidium (Fig. 5 and Supplementary Table 2). The results for NAGase, chitobiosidase and lipase are presented in the same way (Figs. 6, 7, 8 and Supplementary Tables 3–5).

In general, the total elastolytic activity was found to be higher than the equivalent values expressed as activity calculated per single spore and per protein content per single spore. In most cases, activity calculated per protein content per spore equaled those calculated per single spore, suggesting that proteins with elastolytic activity and other proteins were evenly distributed between the spores. However, in C14 (0.1%), C16 (0.0001%), C18 (0.1%), BS (0.0001%) and GO (0.0001%) appeared to vary between spores (Fig. 5; see also Supplementary Table 2).

### NAGase activity in *C. coronatus* conidia

NAGase activity did not differ significantly between SAB and SAB-GM conidia (Fig. 6; see also Table 1 and Supplementary Table 3). Only C16 had a significant effect on total NAGase activity in *C. coronatus* conidia, resulting in elevated NAGase activity at all applied concentrations (0.1–0.0001%; in all cases, *p* < 0.001) The highest increase, sixfold compared to SAB controls, was observed at 0.01% C16. A slight (statistically irrelevant) elevation of total NAGase activity was observed after SAB supplementation with C20 (0.1 and 0.001%).

Similarly to elastase, total NAGase activity tended to be higher than both the activity calculated per protein content per conidium and the NAGase calculated per conidium; both of the latter being equal. Only a couple of exceptions were observed: C16 (0.001% and 0.0001%) and C24 (0.1%) (Fig. 6; see also Supplementary Table 3).

### Chitobiosidase activity of *C. coronatus*

No significant differences were observed between SAB and SAB-GM with regard to total chitobiosidase activity (Fig. 7; see also Table 1 and Supplementary Table 4). Supplementation of SAB with C16 at all concentrations elevated total chitobiosidase activity compared with SAB alone (in all cases, *p* < 0.001). The greatest (7.2-fold) increase was noticed at the concentration of 0.001% C16. Only a few alcohols slightly (i.e. statistically insignificantly) decreased total chitobiosidase activity: C12 (0.01, 0.001 and 0.0001%), C14 (all concentrations), C20 (0.001 and 0.0001%).

The activity of chitobiosidase resembled that of elastase and NAGase: total chitobiosidase activity was higher than activity calculated per protein content per conidium and higher than enzyme activity calculated per conidium: both values calculated per conidium were equal. However, exceptions were observed in the case of: C16 (0.001 and 0.0001%), C18 (0.1%) and C24 (0.1%) suggesting an uneven distribution of chitobiosidase and other proteins in these conidia (Fig. 7; see also Supplementary Table 4).

### Lipase activity in conidia of *C. coronatus*

No significant differences in total lipase activity were observed between SAB and SAB-GM. The CCs demonstrated a wider range of effects on lipase activity than on elastase or the two chitinases (Fig. 8; see also Table 1 and Supplementary Table 5). Increased total lipolytic activity was observed after SAB supplementation with C10 (0.01 and 0.001%; *p* < 0.001 and *p* = 0.005, respectively), C14 (0.1%; *p* = 0.002), C20 (0.01%; *p* < 0.001) and BO (0.001 and 0.0001%; *p* < 0.001 and *p* = 0.016, respectively). Increased lipolytic activity in the spores collected from SAB supplemented with C16 (0.001 and 0.0001%) insignificant in total activity was evident after conversion to one spore or the protein content in one spore.

In contrast, SAB supplementation with 0.01–0.0001% C12 generally completely inhibited lipolytic activity while 0.1% C12 had no impact. A slight, statistically insignificant, decrease in total lipolytic activity was noticed after the addition of C18 (0.0001%), C26 (0.001 and 0.0001%), S (0.0001%) and TA (all concentrations).

The trends described in the case of elastase and two chitinolytic enzymes (NAGase, chitobisidase) were also observed for lipolytic activity: total enzyme activity was higher than activity calculated per protein content per conidium and higher than enzyme activity calculated per conidium, both values being equal. However, exceptions were observed: C16 (at all concentrations) and C24 (0.1%) (Fig. 8; see also Supplementary Table 5).

### Principal component analysis (PCA)

**Figure 9:**
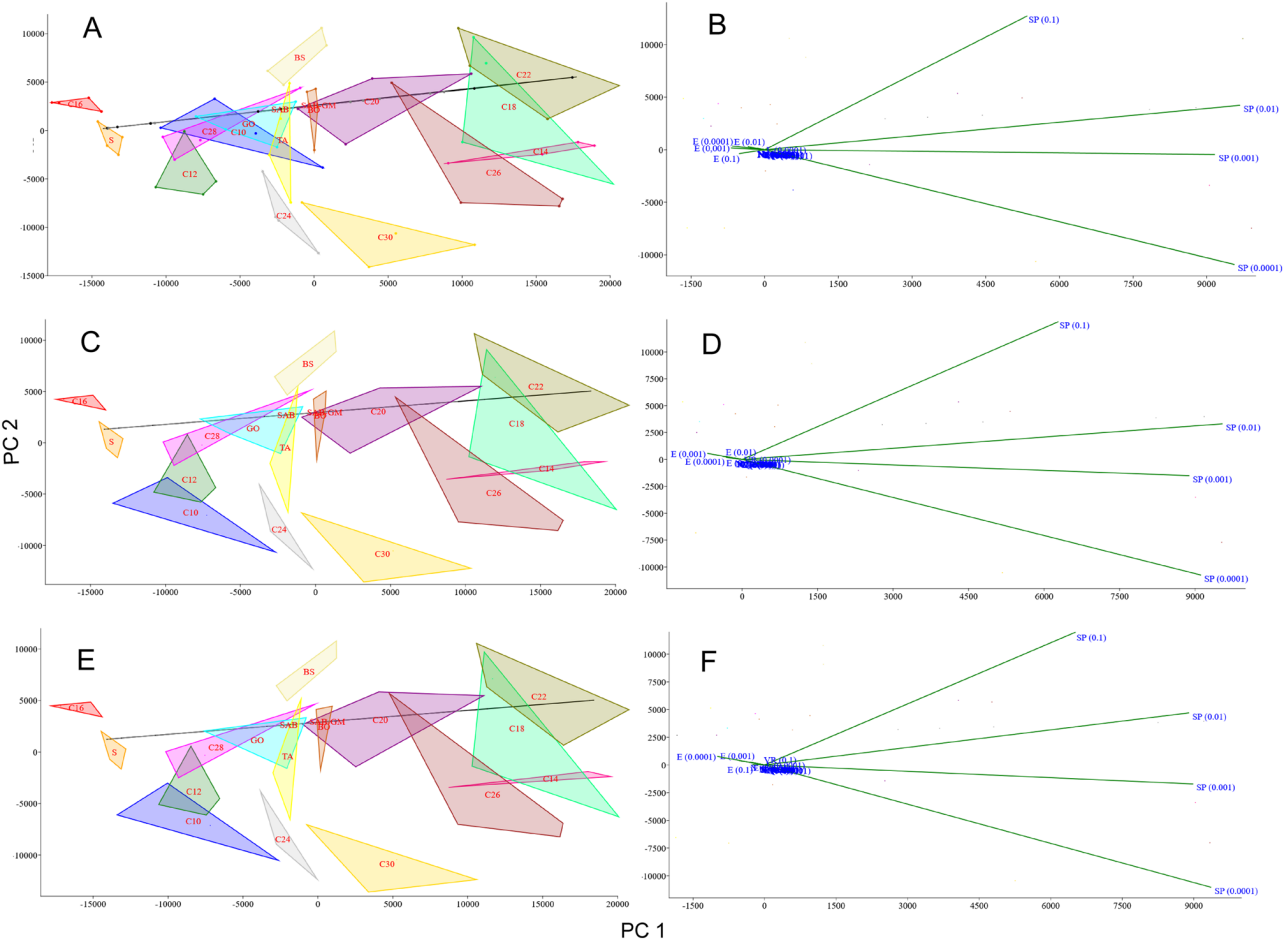
The score (**A**, **C**, **E**) and loading plot (**B**, **D**, **F**) of the principal component analysis (PCA) of variance of conidia activity from SAB medium supplemented with cuticular compounds (CCs) and from control media (SAB and SAB-GM). Enzymatic activity, shown as total activity (**A**, **B**), activity per protein content in one conidium [pM/min/ng of protein] (**C**, **D**) and activity in one conidium [pM/conidium] (**E**, **F**). *C10–C30* fatty alcohols, *BO* butyl oleate, *BS* butyl stearate, *GO* glycerol oleate, *S* squalene, *TA* tocopherol acetate, *SP* sporulation, *VR* virulence, *P* protein content, *E* elastase activity, *N* NAGase activity, *C* chitobiosidase activity, *L* lipase activity; (0.1), (0.01), (0.001), (0.0001)—concentration of CCs added to the SAB medium.

The principal component analysis (PCA, Fig. 9) showed a clear distinction between the effects of CCs on the sporulation of *C. coronatus* and the ability to infect *G. mellonella* larvae. After analysis of all CCs, some parameters overlapped with those of the controls: GO, TA and C28 overlap with SAB, while BO and C20 overlap with SAB-GM. Interestingly, the SAB and SAB-GM parameters do not overlap. However, others were found to significantly differ from controls. This confirms that in most cases, the addition of a CC affected the sporulation of *C. coronatus* and the ability of spores to infect *G. mellonella* larvae. The first component (PC1) explained about 72% of the variation (the exact values depend on the used method of enzymatic activity calculation; Supplementary Table 6), with the values for sporulation (after adding all concentrations of CCs) representing the largest contribution. The second component (PC2) explained about 20% of the variation, with the largest contributions also being from sporulation.

**Figure 10:**
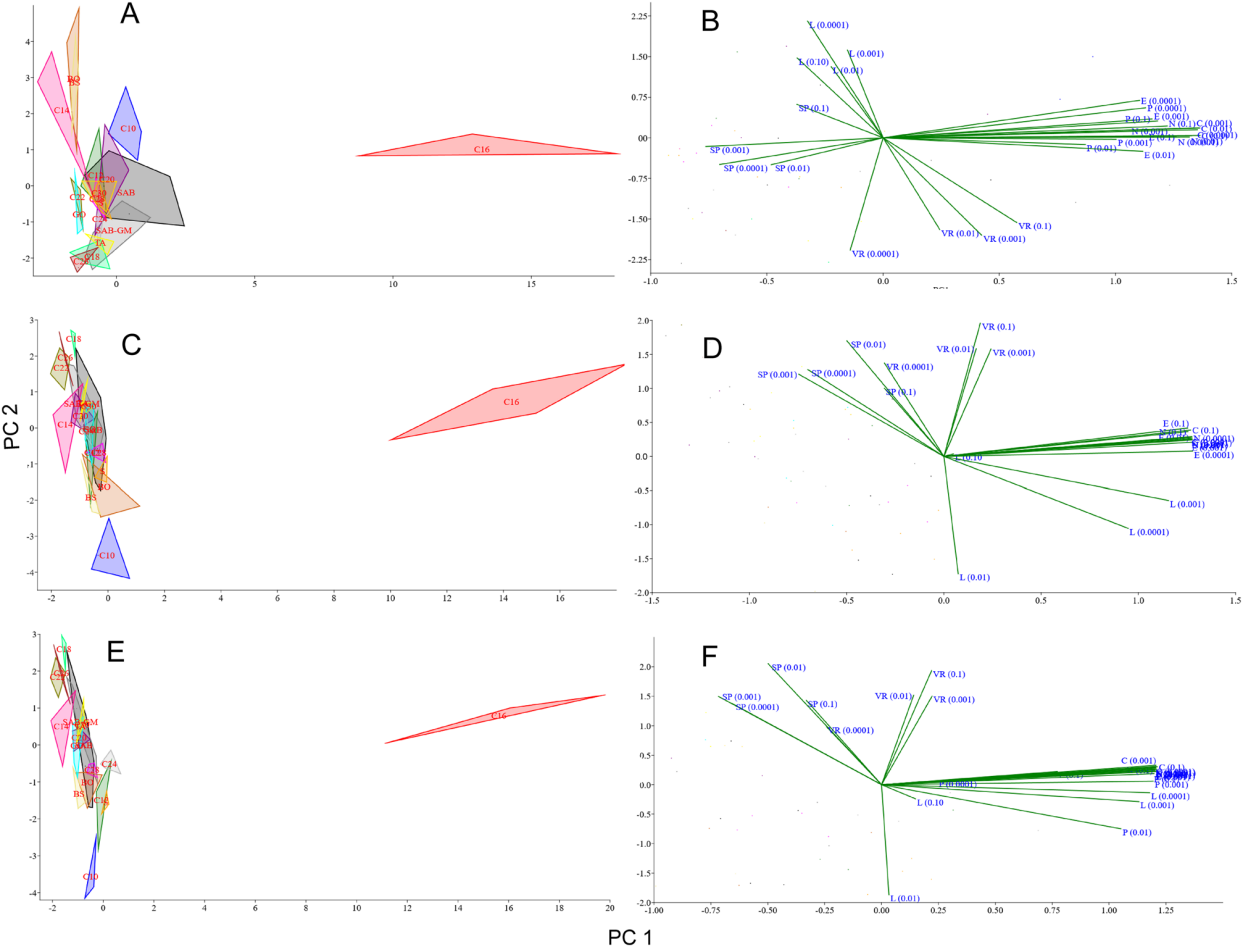
The score (**A**, **C**, **E**) and loading plot (**B**, **D**, **F**) of the principal component analysis (PCA) for correlation between activity of conidia from SAB medium supplemented with cuticular compounds (CCs) and from control media (SAB and SAB-GM). Enzymatic activity, shown as total activity (**A**, **B**), activity per protein content in one conidium [pM/min/ng of protein] (**C**, **D**) and activity in one conidium [pM/conidium] (**E**, **F**). *C10–C30* fatty alcohols, *BO* butyl oleate, *BS* butyl stearate, *GO* glycerol oleate, *S* squalene, *TA* tocopherol acetate, *SP* sporulation, *VR* virulence, *P* protein content, *E* elastase activity, *N* NAGase activity, *C* chitobiosidase activity, *L* lipase activity; (0.1), (0.01), (0.001), (0.0001)—concentration of CCs added to the SAB medium.

The PCA of the correlations between the effects of CCs on the growth and sporulation of *C. coronatus* and its ability to infect the larvae of *G. mellonella* are given in Fig. 10. The sum of its components is dependent on the method used for enzymatic activity calculation. When total enzymatic activity is considered, the first component (PC1) explained 49.26% of the variation, with the largest contribution for elastase, NAGase and chitobiosidase activity, as well as protein content (positive correlation) and sporulation (negative correlation). The second component (PC2) explained 14.92% of the variation, with high contributions from lipase activity (the high positive correlation) and virulency (negative correlation).

The first component (PC1) explained 58.50% of the variation, when the enzymatic activity was calculated per protein content per conidium. The largest contribution was observed for elastase, NAGase and chitobiosidase activity (positive correlations) and sporulation (negative correlation). The second component (PC2) explained 12.81% of the variation, with high contributions made by virulence and sporulation (positive correlation) and lipase activity (negative correlation).

When enzymatic activity is calculated as activity per conidium, PC1 explained the 59.38% of the variation, with the largest contribution observed for elastase, NAGase, chitobiosidase and lipase activity (positive correlations) and sporulation (negative correlation). The second component (PC2) explained 10.91% of the variation, with the high contribution made by virulency and sporulation (positive correlation).

## Discussion

The insect-fungus model is not only beneficial for studies evaluating the use of entomopathogenic fungi to control populations of insect pests, but it may also bring new ideas in the control of mycosis in humans, livestock and domestic animals. The object of our research is the cosmopolitan soil fungus *C. coronatus*, selectively acting entomopathogen capable of infecting also humans, dogs, horses and sheep. *C. coronatus* infects susceptible insect hosts via direct cuticle penetration by invasive hyphae formed after the germination of the spores on the cuticle. Penetration of the host integument is achieved by the mechanical pressure imposed by growing hyphae and the enzymatic degradation of major cuticle components (proteins, chitin and lipids) by proteases, chitinases and lipases produced by the fungus^31,32,40^. Upon invasion of the host hemocoel, hyphae expand but do not infest internal organs due to rapid host death caused by toxic metabolites of the fungus which disorganize functioning of Malpighian tubules, incapacitate immune system and affect serotonin-regulating enzymes^8,10–12,14–16,38^.

The susceptibility or resistance of various insect species to *C. coronatus* invasion results from several factors, including the structure of the host’s exoskeleton and the composition of its cuticle, as well as the efficiency of the host’s immune system^13^. It seems however, that the composition of lipids present in the epicuticle is a key factor in protecting insects against fungal assault^19–26^. Previous studies demonstrating differential efficiency in hydrolyzing in vitro cuticles from larvae of three insect species representing various susceptibilities to *C. coronatus* infection (resistant: *C. vicina*, susceptible: *G. mellonella* and *Dendrolimus pini*) by enzyme cocktail secreted by the fungus into incubation medium, indicate a *C. coronatus* host specificity based on differential cuticle composition^13^. Cuticular free fatty acids (FFAs) profile of *C. vicina* larvae significantly differs from profiles of *D. pini* and *G. mellonella* larvae. The major difference is the presence of FFAs C14:0, C16:1 and C20:0 in the cuticle of *C. vicina* and lack of them in *D. pini*, while larval cuticle of *G. mellonella* contains traces of these FFAs^19^. All these three FFAs when added to *C. coronatus* cultures retarded or totally inhibited fungus growth. Another FFAs effects: decrease of sporulation and virulence (C14:0, C20:0), and lower toxicity of metabolites released into culture medium (C16:1, C20:0) but surprisingly supplementation of culture medium with FFA C16:1 resulted in elevated virulence^30^.

Further experiments proved that the effectiveness of *C. coronatus* enzymes (proteases, chitinases and lipases) hydrolyzing cuticle of several other insect species, both susceptible and resistant to that fungus, is correlated with concentrations of compounds detected in the cuticles of tested insects. Positive correlations indicate compounds used by the fungus as nutrients, whereas negative correlations those engaged in insect resistance^31,33,34^. Our attention was especially drawn to scavenger flies and houseflies, whose larvae and pupae are completely resistant to *C. coronatus* attack. The prompt death of adult flies exposed to *C. coronatus* colonies results from the ingestion of conidia or fungal excretions, as adult flies willingly lick all surfaces, including these covered by fungal spores^31^. Analysis of lipids extracted from the cuticle of *C. vicina*, *C. vomitoria*, *Sarcophaga carnaria* and *Musca domestica* revealed presence of fatty alcohols (C10–C30) and atypical cuticular compounds i.e. butyl oleate (BO), butyl stearate (BS), glycerol oleate (GO), squalene (S), and tocopherol acetate (TA)^23–26^. Fatty alcohols C10–C30 have demonstrated moderate antifungal properties (MIC—Minimal Inhibitory Concentration 256–1024 mg/L) against several entomopathogenic fungal strains (*Beauveria bassiana*, *Lecanicillium lecanii*, *Metarhizium anisopliae*, *Paecilomyces fumosoroseus*, *P. lilacinus*) and very high (MIC 0.512–2 mg/L) against human fungal pathogens (*Aspergillus niger*, *Candida albicans*, *C. lipolytica*, *C. tropicalis*)^22–24^. The antifungal activity of atypical cuticular compounds TA, BS and GO was moderate in both groups of fungal pathogens (MICs 256–1024 mg/L)^25,26^.

The present study examines the impact of these CCs on fungal growth, sporulation, and virulence, as well as activity of elastase, two chitinases (NAGase, chitobiosidase) and lipase in spores produced by *C. coronatus* colonies cultivated on media supplemented with CCs. Our findings indicate that tested CCs have diversified and complex effects on the growth and sporulation of *C. coronatus* and virulence toward the larvae of *G. mellonella.* CCs applied at various concentrations inhibited fungal growth (0.1% C10 only), decreased sporulation (C12, C16, C24, C28, C30, BS, S), virulence (C12, C14, BO, BS) and protein content in conidia (C18). They also reduced activity of cuticle degrading enzymes (CDEs) present in conidia: elastase (C24, BO, BS, S, TA), chitobiosidase (C12, C14, C20) and lipase (C12, C18, C26, S, TA). On the other hand, several CCs enhanced sporulation (C14, C18, C22, C26, C30), virulence (C18, C26, S), conidial protein content (C16, C24, C30, S) and CDE activity: elastase (C10, C16, C18), NAGase (C16, C20), chitobiosidase (C16) and lipase (C10, C14, C16, C20, BO). This variety of action on various elements determining the effectiveness of fungal infection indicates a high degree of complexity of the *C. coronatus* infection strategy. A good example is C10 alcohol, which totally inhibited fungus growth at the maximum concentration of 0.1%, probably by preventing conidia germination, but strongly stimulated the activity of lipase (0.01%) and elastase (0.001 and 0.0001%) at lower concentrations. None of the other tested CCs prevented *C. coronatus* growth, but in *Saccharomyces cerevisiae* C10 and C11 alcohols demonstrated antifungal effects, probably by disrupting native membrane proteins as nonionic surfactants^41^.

Another example of the multilevel effects of the tested substances on the components of the insect destruction system owned by *C. coronatus* is provided by the application of C16 alcohol. Supplementation of culture medium with low concentrations (0.001 and 0.0001%) of C16 alcohol reduced sporulation, but all applied concentrations strongly increased protein content in conidia, as well as the activity of elastase and both chitinases, while lipase activity was elevated to a lesser extent and only at low C16 concentrations (0.001 and 0.0001%). As virulence remained unchanged despite the reduced number of conidia, it appears that the fungus compensated for the quantitative loss in “ammunition” with more heavily-loaded “bullets”. The fact that C10 alcohol stimulated an increase in elastase and lipase activity without increasing the protein content in spores, while C16 alcohol induced elastase and chitinase activity with a simultaneous high accumulation of proteins in the spores demonstrated that the processes responsible for the synthesis and deposition of cuticle-degrading enzymes in conidia are complex and require further detailed research. From our data it appears that cuticle degrading enzymes are not always evenly deposited in the spores produced by the same mycelium. Moreover, their proportions with regard to other conidial proteins varies depending on CC type and concentration in the culture medium, indicating a high complexity of regulatory processes controlling the synthesis of functionally different fungal proteins and their storage in the spores. Considering the fact that single conidium of *C. coronatus* contains from several to even a hundred cell nuclei which are genetically diverse^42,43^, determination of the mechanisms controlling *C. coronatus* genes encoding proteins with proteolytic, chitinolytic and lipolytic activities (226, 299 and 532 genes, respectively^44,45^) is a big challenge.

Two controls were used in the studies assessing the effect of CC on fungal colonies: SAB medium without any additives and SAB enriched with homogenized *G. mellonella* larvae (SAB-GM). The addition of homogenate of whole larvae to SAB is a routine procedure to preserve the virulence of the cultured fungus over subsequent generations^37^. The insect homogenate contains high concentrations of proteins, chitin and lipids, which serve as excellent nutrients for the fungus; therefore, is not surprising that the colonies grown on SAB-GM demonstrated slightly higher spore production with slightly higher virulence, as well as greater protein content, compared with SAB cultures. However, the spores produced by SAB-GM colonies demonstrated lower activity of cuticle-degrading enzymes than those from SAB colonies. Although these differences are clearly visible and frequently seen in previous studies concerning *C. coronatus* grown on media with various nutrient content under optimal and stressful conditions^31,46–48^, they turned out to be statistically insignificant in the current study. Although growth substrates such as the lipids commonly found on insect cuticles generally increase fungal infectivity^49^, conidia derived from media containing lower carbon/nitrogen ratios frequently are found to be more virulent than those harvested from nutrient-rich media^49–51^. Cuticle-degrading enzymes play a key role in determining virulence as they allow the fungus to disrupt the integument of the insect and enter the body cavity. The fungal proteases, chitinases and lipases digesting the major components of insect cuticle demonstrate significantly higher activity in *C. coronatus* cultures propagated in liquid minimal medium (MM) than in those prepared in rich Luria Broth (LB) medium^48^. Furthermore, post-incubation MM is characterized by higher toxicity than LB^8^, partly due to the accumulation of higher amounts of β-carboline alkaloids (harman and norharman); these disorganize the development and immune system of *G. mellonella* larvae by affecting serotonin-regulating enzymes^10,11^. Similarly, basidiomycetes grown in media with low C/N ratios demonstrate reduced mycelial growth but increased production of laccase and other enzymes hydrolyzing lignin and cellulose^52,53^. This common phenomenon results from carbon catabolic repression (CCR) mechanism helping fungi to precisely adapt their physiology to the environment. CCR switches off the enzymes needed to utilize less-favored carbon sources when a more readily available carbon source is present in the medium^54^.

Results indicating that some compounds present in the cuticle of the *C. coronatus*-resistant flies completely inhibit elastase and lipase activity (alcohols: C24 and C12, respectively) or only slightly reduce their activities (squalene and tocopherol acetate) may be a good starting point for the development of new methods of combating conidiobomycosis in humans and animals. Further studies based on other concentrations, vehicles and accompanying substances than those used in the present study, intended to confirm the inhibitory effect of squalene and tocopherol acetate are merited, as these substances demonstrate particular promise. While fatty alcohols can be irritating to human skin, especially the skin of people attacked by the fungus, the therapeutic use of tocopherol acetate and squalene seems safe because these two compounds are natural products of sebocytes and are essential constituents of human sebum^55^.

## Conclusion

Our findings can be used to identify compounds which inhibit or stimulate the development of mycosis, and thus describe the infection strategies employed by *C. coronatus*. The insect-fungus research model is very convenient, as it demonstrates high metabolic flexibility of the fungus and easy adaptation to different media in terms of C and/or N composition or biotic and abiotic stress^56^.

## Supplementary Information

Supplementary Information 1.

Supplementary Information 2.

Supplementary Information 3.

Supplementary Information 4.

Supplementary Information 5.

Supplementary Information 6.

Supplementary Information 7.

Supplementary Information 8.
