# Supplementary material for: Insect cuticular compounds affect Conidiobolus coronatus (Entomopthorales) sporulation and the activity of enzymes involved in fungal infection
Source: Sci Rep. 2022 Aug 10;12:13641. doi: 10.1038/s41598-022-17960-z (PMC9365854; doi:10.1038/s41598-022-17960-z)
Supplement: Supplementary file 1 — Supplementary Information 1. [file 41598_2022_17960_MOESM1_ESM.pdf]

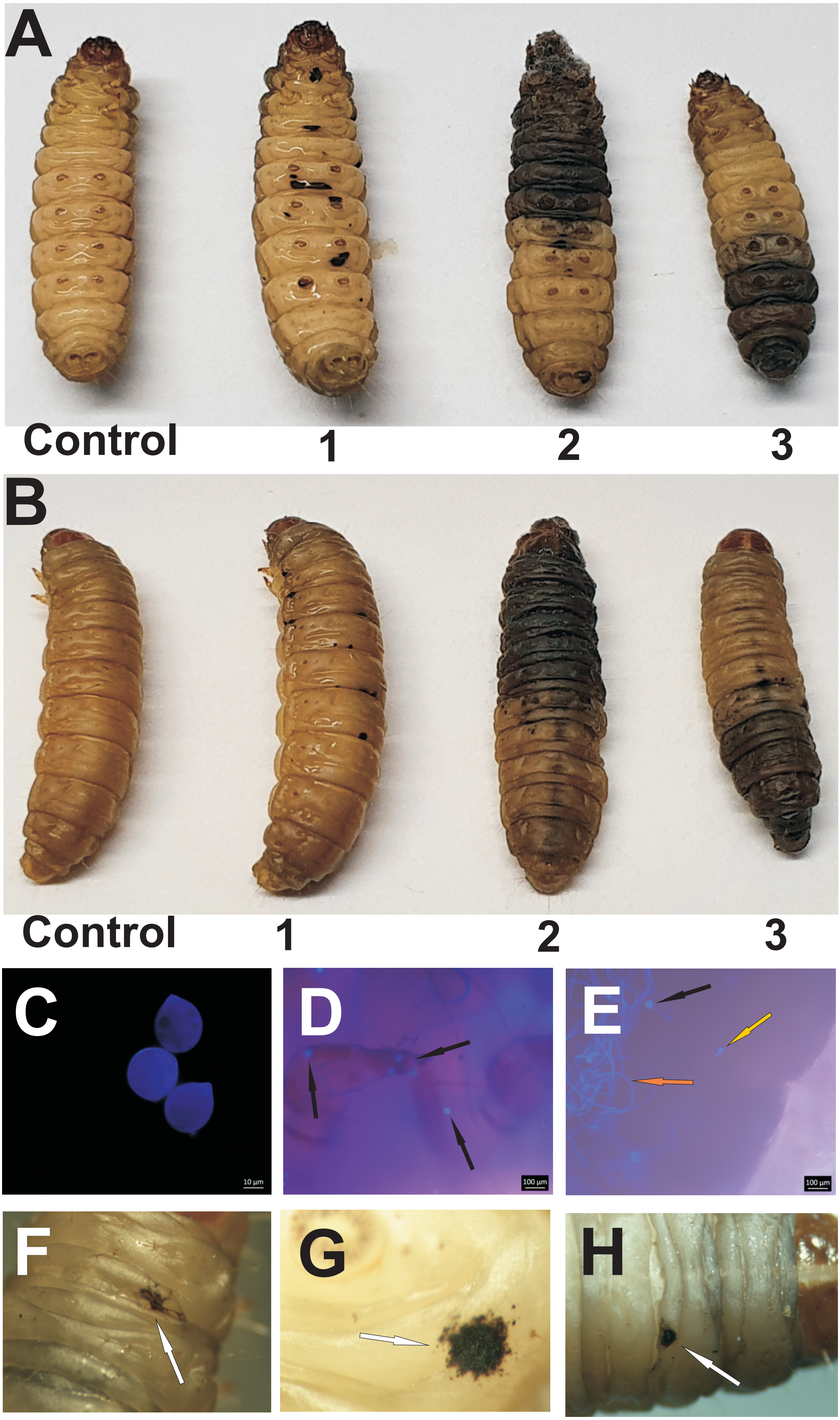
A
Control
1
2
3
B
Control
1
2
3
C
10 µm
D
100 µm
E
100 µm
F
G
H

Supplementary Fig. 1. *Galleria mellonella* larvae infected by *Conidiobolus coronatus.* (A**)** abdominal side view; (B) dorsal side view; (C) conidia washed out from the SAB-GM and stained with Calcofluor White; (D - E) conidia and hyphae present on the cuticle of *G. mellonella* larvae after 20 hours exposure to sporulating SAB-GM fungus culture; (F - H) melanization of the insect cuticle at the penetration site. Control – larva exposed to sterile SAB plate; 1 - 3: successive stages of infection; white arrows: cuticle penetration site; black arrows: conidia attached to the cuticle; yellow arrow: conidium germinating on the cuticle; red arrow: hyphae.
