# Supplementary material for: Insect cuticular compounds affect Conidiobolus coronatus (Entomopthorales) sporulation and the activity of enzymes involved in fungal infection
Source: Sci Rep. 2022 Aug 10;12:13641. doi: 10.1038/s41598-022-17960-z (PMC9365854; doi:10.1038/s41598-022-17960-z)
Supplement: Supplementary file 2 — Supplementary Information 2. [file 41598_2022_17960_MOESM2_ESM.docx]

**Supplementary legends**

Supplementary Figure 1. *Galleria mellonella* larvae infected by *C. coronatus*.

Supplementary Table 1. Protein content in *C. coronatus* conidia.

Supplementary Table 2. Elastase activity in *C. coronatus* conidia.

Supplementary Table 3. NAGase activity in *C. coronatus* conidia.

Supplementary Table 4. Chitobiosidase activity in *C. coronatus* conidia.

Supplementary Table 5. Lipase activity in *C. coronatus* conidia.

Supplementary Table 6. Normalized data for PCA.
