# Supplementary material for: Insect cuticular compounds affect Conidiobolus coronatus (Entomopthorales) sporulation and the activity of enzymes involved in fungal infection
Source: Sci Rep. 2022 Aug 10;12:13641. doi: 10.1038/s41598-022-17960-z (PMC9365854; doi:10.1038/s41598-022-17960-z)
Supplement: Supplementary file 3 — Supplementary Information 3. [file 41598_2022_17960_MOESM3_ESM.pdf]

Supplementary Table 1. Protein content in *C. coronatus* conidia

CC		Total protein content in conidia homogenates		Protein content in one conidium	
		Value (µg/ml)	Average value (µg/ml ± SD)	Value (ng/conidium)	Average value (ng/conidium ± SD)
SAB		63.18	69.98 ± 9.44	0.10	0.11 ± 0.01
		83.07		0.13	
		76.83		0.12	
		78.78		0.12	
		65.52		0.10	
		64.35		0.10	
		58.11		0.09	
SAB-GM		141.96	112.26 ± 21.76	0.19	0.15 ± 0.03
		132.99		0.18	
		88.14		0.12	
		104.52		0.14	
		93.21		0.13	
		96.33		0.13	
		128.7		0.18	
C10	0.1	lack of growth			
	0.01	55.77	59.86 ± 4.29	0.19	0.18 ± 0.01
		63.57		0.16	
		56.55		0.19	
		63.57		0.16	
	0.001	55.38	57.72 ± 7.74	0.09	0.10 ± 0.01
		54.99		0.12	
		51.48		0.09	
		69.03		0.09	
	0.0001	113.10	82.42 ± 27.92	0.09	0.10 ± 0.09
		75.66		0.10	
		58.50		0.11	
C12	0.1	64.35	50.7 ± 9.67	0.20	0.20 ± 0.04
		41.73		0.17	
		47.19		0.19	
		49.53		0.26	
	0.01	49.92	57.62 ± 12.67	0.19	0.22 ± 0.05
		53.82		0.19	
		50.31		0.20	
		76.44		0.29	
	0.001	56.94	56.65 ± 18.23	0.12	0.12 ± 0.04
		65.13		0.15	
		73.32		0.07	
		31.2		0.14	
	0.0001	67.86	47.71 ± 17.69	0.07	0.09 ± 0.01
		40.56		0.09	
		34.71		0.10	
C14	0.1	48.75	60.64 ± 12.82	0.06	0.07 ± 0.02
		78.39		0.10	
		60.84		0.07	
		54.60		0.07	
	0.01	69.81	58.01 ± 12.02	0.07	0.06 ± 0.01
		66.69		0.05	
		45.63		0.07	
		49.92		0.05	
	0.001	140.4	114.95 ± 17.82	0.12	0.10 ± 0.01
		100.23		0.09	
		113.49		0.09	
		105.69		0.09	
	0.0001	169.65	130.75 ± 30.19	0.04	0.05 ± 0.09
		110.76		0.05	
		103.35		0.05	
		139.23		0.06	
C16	0.1	280.60	191.39 ± 74.57	0.88	0.60 ± 0.23
		98.08		0.31	
		195.58		0.62	
		191.29		0.60	
	0.01	203.77	173.61 ± 55.99	0.65	0.56 ± 0.18
		109.00		0.35	
		208.06		0.67	
	0.001	252.52	183.59 ± 54.10	0.68	1.02 ± 0.30
		165.55		0.92	
		192.85		1.40	
		123.43		1.07	
	0.0001	235.36	238.68 ± 31.93	0.56	0.73 ± 0.13
		278.26		0.74	
		240.82		0.77	
		200.26		0.87	
C18	0.1	26.52	29.05 ± 7.57	0.25	0.25 ± 0.10
		38.22		0.25	
		31.2		0.37	
		20.28		0.13	
	0.01	39.78	40.36 ± 12.66	0.05	0.03 ± 0.01
		54.99		0.03	
		42.51		0.04	
		24.18		0.02	
	0.001	47.19	58.30 ± 13.48	0.04	0.05 ± 0.01
		77.22		0.05	
		58.5		0.04	
		50.31		0.07	
	0.0001	51.09	45.24 ± 17.85	0.06	0.08 ± 0.01
		47.19		0.08	
		62.4		0.08	
		20.28		0.09	

C20	0.1	71.76	81.21 ± 9.71	0.10	0.12 ± 0.01
		78.39		0.11	
		79.95		0.14	
		94.77		0.11	
	0.01	45.63	56.35 ± 12.35	0.60	0.07 ± 0.01
		56.16		0.06	
		73.71		0.07	
		49.92		0.09	
	0.001	79.95	58.69 ± 18.99	0.01	0.01 ± 0.00
		62.79		0.09	
		58.11		0.01	
		33.93		0.01	
	0.0001	98.67	95.16 ± 13.76	0.07	0.09 ± 0.02
		113.10		0.09	
		85.80		0.10	
		83.07		0.11	
C22	0.1	82.68	83.07 ± 11.56	0.07	0.07 ± 0.01
		70.20		0.06	
		81.12		0.07	
		98.28		0.09	
	0.01	123.63	95.26 ± 27.59	0.08	0.09 ± 0.03
		111.15		0.12	
		84.24		0.06	
		62.01		0.11	
	0.001	97.11	100.23 ± 23.13	0.09	0.09 ± 0.02
		133.77		0.12	
		83.07		0.08	
		86.97		0.08	
	0.0001	114.66	104.71 ± 12.09	0.05	0.06 ± 0.01
		96.72		0.06	
		115.44		0.06	
		92.04		0.07	
C24	0.1	74.10	55.57 ± 14.33	0.68	0.63 ± 0.16
		44.46		0.51	
		44.07		0.84	
		59.67		0.50	
	0.01	100.62	106.37 ± 5.49	0.30	0.24 ± 0.01
		112.32		0.23	
		102.96		0.26	
		109.59		0.25	
	0.001	130.26	107.44 ± 16.96	0.17	0.14 ± 0.02
		97.50		0.13	
		109.98		0.14	
		92.04		0.12	
	0.0001	90.48	88.14 ± 24.91	0.05	0.06 ± 0.01
		117.00		0.06	
		88.92		0.07	
C26	0.1	52.26	40.07 ± 8.48	0.10	0.10 ± 0.02
		36.66		0.08	
		32.76		0.13	
		38.61		0.09	
	0.01	164.97	102.37 ± 42.77	0.14	0.08 ± 0.03
		88.53		0.07	
		68.25		0.06	
		87.75		0.07	
	0.001	108.81	84.04 ± 23.44	0.10	0.08 ± 0.02
		87.75		0.09	
		52.26		0.05	
		87.36		0.09	
	0.0001	74.49	71.76 ± 18.28	0.04	0.06 ± 0.01
		85.80		0.06	
		81.51		0.06	
		45.24		0.07	
C28	0.1	70.39	54.89 ± 13.04	0.09	0.13 ± 0.03
		49.72		0.11	
		39.97		0.14	
		59.47		0.16	
	0.01	89.11	55.28 ± 24.70 zł	0.09	0.15 ± 0.07
		41.14		0.11	
		57.52		0.16	
		33.34		0.24	
	0.001	93.79	81.9 ± 25.83	0.17	0.15 ± 0.05
		99.25		0.18	
		43.48		0.08	
		91.06		0.17	
	0.0001	77.41	73.22 ± 22.40	0.10	0.13 ± 0.02
		58.30		0.13	
		103.15		0.13	
		54.01		0.15	
C30	0.1	74.88	60.45 ± 25.26	0.34	0.27 ± 0.11
		63.96		0.11	
		79.17		0.36	
		23.79		0.29	
	0.01	53.04	42.02 ± 14.02	0.09	0.07 ± 0.02
		53.04		0.07	
		23.79		0.04	
		38.22		0.09	
	0.001	39.78	58.89 ± 26.11	0.05	0.07 ± 0.03
		97.5		0.11	
		49.14		0.06	
		49.14		0.06	
	0.0001	79.95	58.11 ± 17.83	0.04	0.05 ± 0.01
		48.75		0.05	
		39.39		0.00	
		64.35		0.06	

**BO**	0.1	78.78<br>92.82<br>70.20<br>82.29	81.02 ±9.36	0.11<br>0.12<br>0.13<br>0.10	0.11 ± 0.01
	0.01	94.77<br>64.35<br>67.47<br>79.17	76.44 ±13.78	0.11<br>0.13<br>0.11<br>0.16	0.13 ± 0.02
	0.001	60.84<br>46.80<br>61.62<br>53.82	55.77 ±6.932791646	0.09<br>0.08<br>0.09<br>0.07	0.09 ± 0.01
	0.0001	66.30<br>52.65<br>63.96<br>88.53	67.86 ±15.01	0.07<br>0.09<br>0.09<br>0.11	0.09 ± 0.02
**BS**	0.1	34.71<br>47.97<br>40.17<br>89.70	53.14 ±24.97	0.07<br>0.06<br>0.05<br>0.12	0.07 ± 0.03
	0.01	85.41<br>63.57<br>82.68<br>81.12	78.19 ±9.91	0.12<br>0.09<br>0.11<br>0.11	0.11 ± 0.01
	0.001	54.99<br>81.12<br>67.08<br>40.95	61.03 ±17.12	0.08<br>0.07<br>0.10<br>0.05	0.07 ± 0.02
	0.0001	95.55<br>87.75<br>96.33<br>78.00	89.41 ±8.53	0.06<br>0.07<br>0.08<br>0.09	0.07 ± 0.01
**GO**	0.1	38.22<br>55.38<br>33.93<br>32.37	39.97 ±10.56	0.07<br>0.06<br>0.06<br>0.10	0.07 ± 0.02
	0.01	31.59<br>36.27<br>52.26<br>35.49	38.90 ±9.14	0.07<br>0.08<br>0.08<br>0.12	0.09 ± 0.02
	0.001	44.46<br>35.88<br>32.37<br>51.48	41.04 ±8.61	0.06<br>0.08<br>0.06<br>0.09	0.07 ± 0.02
	0.0001	40.17<br>34.71<br>45.63<br>45.24	41.44 ±5.13	0.08<br>0.10<br>0.11<br>0.12	0.10 ± 0.02
**S**	0.1	112.71<br>88.53<br>90.09<br>83.46	93.70 ±12.99	0.49<br>0.39<br>0.39<br>0.36	0.41 ± 0.06
	0.01	76.05<br>49.14<br>67.86<br>65.13	64.54 ±11.27	0.13<br>0.17<br>0.18<br>0.20	0.17 ± 0.03
	0.001	61.23<br>72.93<br>60.45<br>50.70	61.33 ±9.10 zł	0.19<br>0.23<br>0.19<br>0.16	0.19 ± 0.03
	0.0001	88.92<br>66.69<br>59.67<br>82.68	74.49 ±13.61	0.16<br>0.21<br>0.22<br>0.25	0.21 ± 0.04
**TA**	0.1	75.27<br>53.04<br>83.07<br>106.86	79.56 ±22.21	0.11<br>0.16<br>0.18<br>0.23	0.17 ± 0.05
	0.01	63.57<br>53.04<br>130.26<br>54.99	75.46 ±36.81	0.11<br>0.09<br>0.22<br>0.09	0.13 ± 0.06
	0.001	79.56<br>59.67<br>60.06<br>69.03	67.08 ±9.37	0.11<br>0.12<br>0.11<br>0.14	0.12 ± 0.02
	0.0001	93.60<br>62.79<br>60.06<br>101.79	79.56 ±21.23	0.07<br>0.09<br>0.09<br>0.10	0.09 ± 0.01

CC – cuticular compound; SD – standard deviation; SAB – *C. coronatus* colonies cultivated on Sabouraud agar medium; SAB-GM – *C. coronatus* colonies cultivated on Sabouraud agar medium with the addition of homogenized *G. mellonella* larvae; C10-C30 – fatty alcohols; BO – butyl oleate; BS – butyl stearate; GO – glycerol oleate; S – squalene; TA – tocopherol acetate
