# Supplementary material for: Insect cuticular compounds affect Conidiobolus coronatus (Entomopthorales) sporulation and the activity of enzymes involved in fungal infection
Source: Sci Rep. 2022 Aug 10;12:13641. doi: 10.1038/s41598-022-17960-z (PMC9365854; doi:10.1038/s41598-022-17960-z)
Supplement: Supplementary file 4 — Supplementary Information 4. [file 41598_2022_17960_MOESM4_ESM.pdf]

Supplementary Table 2. Elastase activity in *C. coronatus* conidia

CC		Total activity		Activity per protein content in one conidium		Activity in one conidium	
		Value (pM/min/ng)	Average value (pM/min/ng ± SD)	Value (pM/min/ng)	Average value (pM/min/ng ± SD)	Value (pM/min/conidium)	Average value (pM/conidium ± SD)
SAB		271.34	600.82 ± 472.24	29.13	64.50 ± 50.69	29.13	64.50 ± 50.69
		253.25		27.19		27.19	
		185.41		19.91		19.91	
		212.55		22.82		22.82	
		927.06		99.52		99.52	
		1257.19		134.96		134.96	
		1098.91		117.97		117.97	
SAB-GM		90.20	80.25 ± 73.90	13.90	12.37 ± 11.39	13.90	10.30 ± 12.68
		233.96		36.06		36.05	
		90.20		13.90		13.90	
		11.27		1.74		1.74	
		60.47		9.32		3.36	
		47.43		7.31		2.72	
		28.19		4.34		0.43	
C10	0.1	lack of growth					
	0.01	134.94	415.61 ± 243.11	24.21	74.57 ± 43.62	23.18	71.40 ± 41.77
		291.46		52.30		50.08	
		593.72		106.53		102.01	
		642.30		115.25		110.35	
	0.001	4111.95	2933.19 ± 2283.19	404.03	288.21 ± 224.34	404.03	288.21 ± 224.34
		301.54		29.63		29.63	
		4386.08		430.96		430.96	
	0.0001	3087.37	2740.04 ± 420.40	264.83	235.04 ± 36.06	370.94	329.21 ± 50.51
		2238.35		192.00		268.93	
		3087.37		264.83		370.94	
		2547.08		218.49		306.02	
C12	0.1	1048.61	880.08 ± 320.75	211.76	177.72 ± 64.77	211.76	177.72 ± 64.77
		511.82		103.36		103.36	
		1229.62		248.31		248.32	
		730.28		147.47		147.47	
	0.01	585.46	856.62 ± 392.34	128.15	187.50 ± 85.88	114.20	167.09 ± 76.53
		677.90		148.38		132.23	
		1306.50		285.98		254.85	
	0.001	134.07	202.51 ± 71.01	16.06	24.26 ± 8.51	16.06	24.26 ± 8.51
		301.66		36.14		36.14	
		195.52		23.42		23.42	
		178.76		21.41		21.41	
	0.0001	729.41	512.82 ± 190.18	62.79	44.15 ± 0.55	49.34	34.69 ± 12.87
		435.97		37.53		29.49	
		373.09		32.12		25.24	
C14	0.1	156.54	135.67 ± 27.61	11.70	10.14 ± 2.06	11.70	56.92 ± 79.01
		146.11		10.92		10.92	
		104.36		7.80		148.15	
	0.01	73.00	157.68 ± 94.05	4.42	9.55 ± 5.70	4.62	9.99 ± 5.96
		291.90		17.68		18.50	
		125.10		7.58		7.93	
		140.74		8.53		8.92	
	0.001	517.55	226.66 ± 253.57	5.14	7.09 ± 3.33	5.14	7.09 ± 3.33
		52.30		5.19		5.19	
		110.12		10.93		10.93	
	0.0001	79.87	96.82 ± 11.53	3.85	4.66 ± 0.55	7.79	9.44 ± 1.12
		104.08		5.01		10.15	
		99.24		4.78		9.68	
		104.08		5.01		10.15	
C16	0.1	4221.23	2999.75 ± 1593.37	2549.62	1811.85 ± 962.39	2549.62	1811.85 ± 962.39
		1211.97		732.03		732.03	
		4459.32		2693.43		2693.43	
		2106.48		1272.31		1272.31	
	0.01	4901.36	2985.65 ± 2051.21	2727.40	1661.39 ± 1141.41	2727.40	1661.39 ± 1141.41
		1410.80		785.05		785.05	
		1024.38		570.02		570.02	
		4606.07		2563.09		2563.09	
	0.001	4033.42	3633.96 ± 1292.89	4036.33	3636.58 ± 1293.82	4105.37	3698.78 ± 1315.95
		1711.62		1712.85		1742.15	
		4404.01		4407.19		4482.57	
		4386.78		4389.94		4465.03	
	0.0001	2296.38	2517.14 ± 691.28	6246.17	6846.62 ±1880.29	6246.17	6846.62 ± 1880.29
		1691.79		4601.68		4601.68	
		2760.43		7508.38		7508.38	
		3319.95		9030.26		9030.26	
C18	0.1	446.55	465.62 ± 111.91	111.67	116.43 ± 27.98	16.95	17.67 ± 4.25
		566.36		141.63		21.50	
		533.69		133.46		20.26	
		315.86		78.98		11.99	
	0.01	2704.75	3334.55 ± 754.51	93.90	115.76 ± 26.19	93.90	115.76 ± 26.19
		3128.10		108.60		108.60	
		4170.80		144.80		144.80	
	0.001	282.23	811.42 ± 470.28	14.42	41.47 ± 24.04	14.42	41.47 ± 24.04
		879.27		44.94		44.94	
		673.02		34.40		34.40	
		1411.17		72.13		72.13	
	0.0001	440.69	438.35 ± 94.45	35.36	35.17 ± 7.58	24.78	24.65 ± 5.31
		342.76		27.50		19.28	
		531.62		42.66		29.90	

C20	0.1	81.82<br>85.72<br>155.85	107.80 ± 41.66	9.53<br>9.99<br>18.16	12.56 ± 4.85	9.53<br>9.99<br>18.16	12.56 ± 4.85
	0.01	196.54<br>269.54<br>61.77	175.95 ± 105.40	13.85<br>19.00<br>4.35	12.40 ± 7.43	13.85<br>19.00<br>4.35	12.40 ± 7.43
	0.001	183.31<br>134.79<br>157.43	158.51 ± 34.31	12.36<br>9.09<br>106.19	42.55 ± 55.14	59.65<br>43.86<br>51.23	51.58 ± 7.90
	0.0001	309.27<br>242.76<br>212.83<br>525.43	322.57 ± 141.11	28.34<br>22.24<br>19.50<br>48.14	29.56 ± 12.93	41.78<br>32.79<br>28.75<br>70.98	43.57 ± 19.06
C22	0.1	125.27<br>153.74<br>153.74<br>204.99	159.44 ± 33.20	9.32<br>11.44<br>11.44<br>15.25	11.86 ± 2.47	6.24<br>7.65<br>7.65<br>10.20	7.94 ± 1.65
	0.01	107.10<br>124.95<br>98.17	110.07 ± 13.63	9.80<br>11.43<br>8.98	10.07 ± 1.25	10.94<br>12.77<br>10.03	11.25 ± 1.39
	0.001	91.30<br>279.80<br>126.65<br>241.51	184.82 ± 90.12	8.44<br>25.85<br>11.70<br>22.32	17.08 ± 8.33	9.04<br>27.72<br>12.54<br>23.92	18.31 ± 8.93
	0.0001	118.48<br>161.57<br>183.11<br>254.92	179.52 ± 56.99	7.35<br>10.02<br>11.36<br>15.81	11.13 ± 3.53	10.03<br>13.68<br>15.51<br>21.59	15.20 ± 4.83
C24	0.1	lack of activity					
	0.01	182.71<br>252.48<br>119.59<br>298.99	213.44 ± 78.73	44.29<br>61.22<br>28.99<br>72.49	51.75 ± 19.09	39.67<br>54.82<br>25.97<br>64.91	46.34 ± 17.09
	0.001	91.56<br>113.66<br>262.05<br>350.46	204.43 ± 123.32	12.57<br>15.61<br>35.98<br>48.12	28.07 ± 16.93	11.73<br>14.56<br>33.57<br>44.89	26.19 ± 15.80
	0.0001	135.99<br>356.60<br>247.81<br>335.45	268.96 ± 100.38	6.13<br>16.06<br>11.16<br>15.11	12.12 ± 4.52	14.09<br>36.94<br>25.67<br>34.75	27.86 ± 10.40
C26	0.1	315.89<br>244.81<br>1160.87<br>576.49	574.51 ± 416.09	32.46<br>25.15<br>119.28<br>59.23	59.03 ± 42.75	32.46<br>25.15<br>119.28<br>59.23	59.03 ± 42.75
	0.01	46.37<br>129.83<br>61.82<br>120.55	89.64 ± 41.70	3.94<br>11.04<br>5.26<br>10.25	7.62 ± 3.54	3.94<br>11.04<br>5.26<br>10.25	7.62 ± 3.54
	0.001	71.54<br>109.19<br>143.08<br>169.44	123.31 ± 42.42	5.90<br>9.01<br>11.80<br>13.98	10.17 ± 3.50	5.90<br>9.01<br>11.80<br>13.98	10.17 ± 3.50
	0.0001	317.51<br>110.25<br>498.32<br>211.68	284.44 ± 165.81	17.93<br>6.22<br>28.14<br>11.95	16.06 ± 9.36	19.93<br>6.92<br>31.28<br>13.29	17.85 ± 10.41
C28	0.1	109.53<br>167.18<br>219.07<br>259.42	188.80 ± 64.95	13.94<br>21.27<br>27.87<br>33.01	24.02 ± 8.26	13.94<br>21.27<br>27.87<br>33.01	24.02 ± 8.26
	0.01	412.15<br>646.85<br>274.77	444.59 ± 188.15	62.32<br>97.80<br>41.54	67.22 ± 28.45	62.32<br>97.80<br>41.54	67.22 ± 28.45
	0.001	224.11<br>714.83<br>227.97<br>289.79	364.17 ± 235.70	34.07<br>108.68<br>34.66<br>44.06	55.37 ± 35.83	34.07<br>108.68<br>34.66<br>44.06	55.37 ± 35.83
	0.0001	376.00<br>242.02<br>587.77<br>432.18	409.49 ± 143.14	47.41<br>30.52<br>74.11<br>54.50	51.64 ± 18.05	53.78<br>34.62<br>84.08<br>61.82	58.58 ± 20.47
C30	0.1	1455.33<br>711.96<br>675.31<br>1272.10	1028.68 ± 394.32	401.02<br>196.18<br>186.09<br>350.53	283.46 ± 108.66	401.02<br>196.18<br>186.09<br>350.53	283.46 ± 108.66
	0.01	655.16<br>421.71<br>1024.16<br>753.06	713.53 ± 249.41	47.66<br>30.68<br>74.50<br>54.78	51.90 ± 18.14	47.66<br>30.68<br>74.50<br>54.78	51.90 ± 18.14
	0.001	1117.72<br>209.57<br>1069.36<br>1434.77	957.86 ± 524.52	75.85<br>14.22<br>72.57<br>97.37	65.00 ± 35.60	75.85<br>14.22<br>72.57<br>97.37	65.00 ± 35.60
	0.0001	729.74<br>168.82<br>370.31<br>506.46	443.83 ± 235.73	36.46<br>8.43<br>18.50<br>25.30	22.17 ± 11.78	32.82<br>7.59<br>16.66<br>22.78	19.96 ± 10.60

**BO**	0.1	64.44<br>111.31<br>48.82<br>44.92	67.37 ± 30.48	7.44<br>12.85<br>5.63<br>5.18	7.78 ± 3.52	7.44<br>12.85<br>5.63<br>5.18	7.78 ± 3.52
	0.01	47.61<br>47.61<br>39.33<br>93.15	56.92 ± 24.46	6.04<br>6.04<br>4.99<br>11.83	7.23 ± 3.10	6.04<br>6.04<br>4.99<br>11.83	7.23 ± 3.10
	0.001	99.30<br>49.25<br>69.99<br>75.18	73.43 ± 20.56	8.57<br>4.25<br>6.04<br>6.49	6.34 ± 1.77	8.57<br>4.65<br>6.61<br>7.10	6.74 ± 1.62
	0.0001	854.70<br>571.76<br>442.09<br>1081.64	737.55 ± 286.88	76.72<br>51.32<br>39.68<br>97.09	66.20 ± 25.75	80.66<br>53.96<br>41.72<br>102.08	69.60 ± 27.07
**BS**	0.1	44.66<br>50.62<br>98.26<br>122.08	78.91 ± 37.47	3.30<br>3.74<br>7.26<br>9.02	5.83 ± 2.77	3.30<br>3.74<br>7.26<br>9.02	5.83 ± 2.77
	0.01	58.68<br>58.68<br>54.63	57.33 ± 2.34	6.21<br>6.21<br>5.78	6.07 ± 0.25	6.21<br>6.21<br>5.78	6.07 ± 0.25
	0.001	137.40<br>178.88<br>173.69<br>85.55	143.88 ± 43.04	10.15<br>13.21<br>12.83<br>6.32	10.63 ± 3.18	10.15<br>13.21<br>12.83<br>6.32	10.63 ± 3.18
	0.0001	548.05<br>503.31<br>552.53<br>447.39	512.82 ± 48.95	40.20<br>36.92<br>40.53<br>32.82	37.62 ± 3.59	55.68<br>51.14<br>56.14<br>45.46	52.10 ± 4.97
**GO**	0.1	83.12<br>281.03<br>114.79<br>51.46	132.60 ± 102.28	5.93<br>20.04<br>8.18<br>3.67	9.45 ± 7.29	5.93<br>20.04<br>8.18<br>3.67	9.45 ± 7.29
	0.01	117.95<br>215.57<br>52.87	128.80 ± 81.89	10.63<br>19.44<br>4.77	11.61 ± 7.38	10.63<br>19.44<br>4.77	11.61 ± 7.38
	0.001	80.95<br>88.66<br>188.88	119.50 ± 60.21	6.06<br>6.63<br>14.14	8.94 ± 4.51	6.06<br>6.63<br>14.14	8.94 ± 4.51
	0.0001	497.13<br>429.56<br>564.70<br>559.88	512.82 ± 63.47	51.42<br>44.43<br>58.41<br>57.91	53.04 ± 4.21	33.01<br>28.52<br>37.50<br>37.18	34.05 ± 4.21
**S**	0.1	92.88<br>96.26<br>136.78<br>278.64	151.14 ± 87.31	37.98<br>39.36<br>55.94<br>113.94	61.81 ± 35.70	37.98<br>39.36<br>55.94<br>113.94	61.81 ± 35.70
	0.01	71.09<br>17.16<br>85.80<br>36.77	52.70 ± 31.36	11.99<br>2.89<br>14.47<br>6.20	8.89 ± 5.29	11.99<br>2.89<br>14.47<br>6.20	8.89 ± 5.29
	0.001	95.46<br>64.50<br>121.26<br>245.10	131.58 ± 79.16	18.33<br>12.39<br>23.29<br>47.07	25.27 ± 15.20	18.33<br>12.39<br>23.29<br>47.07	25.27 ± 15.20
	0.0001	612.16<br>459.12<br>410.79<br>569.20	512.82 ± 93.70	127.64<br>95.73<br>85.65<br>118.68	106.93 ± 19.54	147.30<br>110.48<br>98.85<br>136.97	123.40 ± 22.55
**TA**	0.1	59.66<br>79.55<br>59.66<br>55.69	63.64 ± 10.77	10.19<br>13.59<br>10.19<br>9.52	10.87 ± 1.84	10.19<br>13.59<br>10.19<br>9.52	10.87 ± 1.84
	0.01	67.09<br>29.35<br>75.48<br>79.67	62.90 ± 22.97	8.48<br>3.71<br>9.54<br>10.07	7.95 ± 2.90	8.48<br>3.71<br>9.54<br>10.07	7.95 ± 2.90
	0.001	500.06<br>146.24<br>259.47	301.92 ± 180.69	59.57<br>17.42<br>30.91	35.97 ± 21.53	59.57<br>17.42<br>30.91	35.97 ± 21.53
	0.0001	603.32<br>404.72<br>387.13<br>656.11	512.82 ± 136.87	53.79<br>36.08<br>34.52<br>58.50	45.72 ± 12.20	66.30<br>44.48<br>42.54<br>72.10	56.36 ± 15.04

CC – cuticular compound; SD – standard deviation; SAB – *C. coronatus* colonies cultivated on Sabouraud agar medium; SAB-GM – *C. coronatus* colonies cultivated on Sabouraud agar medium with the addition of homogenized *G. mellonella* larvae; C10-C30 – fatty alcohols; BO – butyl oleate; BS – butyl stearate; GO – glycerol oleate; S – squalene; TA – tocopherol acetate
