# Supplementary material for: Insect cuticular compounds affect Conidiobolus coronatus (Entomopthorales) sporulation and the activity of enzymes involved in fungal infection
Source: Sci Rep. 2022 Aug 10;12:13641. doi: 10.1038/s41598-022-17960-z (PMC9365854; doi:10.1038/s41598-022-17960-z)
Supplement: Supplementary file 5 — Supplementary Information 5. [file 41598_2022_17960_MOESM5_ESM.pdf]

Supplementary Table 3. NAGase activity in *C. coronatus* conidia

CC		Total activity		Activity per protein content in one conidium		Activity in one conidium	
		Value (pM/min/ng)	Average value (pM/min/ng ± SD)	Value (pM/min/ng)	Average value (pM/min/ng ± SD)	Value (pM/min/conidium)	Average value (pM/conidium ± SD)
SAB		36.19	154.46 ± 132.95	3.88	16.58 ± 14.27	3.88	16.58 ± 14.27
		65.60		7.04		7.04	
		42.98		4.61		4.61	
		49.76		5.34		5.34	
		309.9		33.27		33.27	
		307.63		33.02		33.02	
		269.18		28.90		28.90	
SAB-GM		38.07	48.15 ± 35.09	5.87	7.42 ± 5.41	5.97	7.55 ± 5.50
		77.56		11.95		12.16	
		36.60		5.65		5.75	
		114.23		17.60		17.92	
		31.02		4.78		4.87	
		22.56		3.48		3.54	
		16.92		2.61		2.65	
C10	0.1	lack of growth					
	0.01	59.42	66.62 ± 17.36	10.66	11.95 ± 3.11	10.21	11.45 ± 2.98
		54.02		9.69		9.28	
		86.43		15.51		14.85	
	0.001	166.00	123.91 ± 68.88	16.31	12.17 ± 3.62	16.31	12.17 ± 3.62
		108.37		10.65		10.65	
		97.39		9.57		9.57	
	0.0001	103.32	75.64 ± 23.99	8.86	6.49 ± 2.06	12.41	9.09 ± 2.88
		62.76		5.38		7.54	
		60.83		5.22		7.31	
C12	0.1	349.81	195.99 ± 113.31	70.64	39.58 ± 22.88	70.64	39.58 ± 22.88
		78.08		15.77		15.77	
		165.54		33.43		33.43	
		190.52		38.47		38.47	
	0.01	72.47	92.00 ± 36.53	15.86	20.14 ± 8.00	14.13	17.94 ± 7.12
		69.39		15.19		13.53	
		134.14		29.36		26.17	
	0.001	48.92	71.98 ± 50.08	5.86	8.62 ± 6.00	5.86	5.64 ± 0.70
		51.71		6.19		6.19	
		40.50		4.85		4.85	
		146.76		17.58			
	0.0001	34.85	34.02 ± 3.18	3.00	2.93 ± 0.27	2.36	2.30 ± 0.21
		31.53		2.71		2.13	
		31.53		2.71		2.13	
		38.17		3.29		2.58	
C14	0.1	194.53	113.58 ± 57.44	14.53	8.49 ± 4.29	14.53	8.49 ± 4.29
		58.75		4.39		4.39	
		100.53		7.51		7.51	
		100.30		7.51		7.51	
	0.01	61.30	66.51 ± 11.37	3.71	4.03 ± 0.69	3.88	4.21 ± 0.72
		58.69		3.55		3.72	
		79.55		4.82		5.04	
	0.001	30.99	16.19 ± 11.87	3.08	1.61 ± 1.18	3.08	1.50 ± 1.44
		14.46		1.44		0.20	
		17.22		1.71		1.40	
		2.07		0.20			
	0.0001	10.29	6.26 ± 4.25	0.50	0.23 ± 0.00	1.00	0.61 ± 0.41
		1.82		0.09		0.82	
		6.66		0.32		0.65	
C16	0.1	1387.5	879.50 ± 551.48	838.05	531.22 ± 333.09	838.05	531.22 ± 333.09
		635.42		383.79		383.79	
		1276.64		771.09		771.09	
		218.43		131.93		131.93	
	0.01	1619.42	938.32 ± 645.52	901.14	522.13 ± 359.21	901.14	522.13 ± 359.21
		1302.92		725.02		725.02	
		646.22		359.59		359.59	
		184.70		102.78		102.78	
	0.001	1130.34	732.50 ± 516.81	1131.15	733.03 ± 517.18	1150.50	745.56 ± 526.03
		381.67		381.94		388.47	
		199.67		199.82		203.24	
		1218.32		1219.19		1240.05	
	0.0001	878.08	761.64 ± 203.54	645.88	560.23 ± 149.72	2388.38	2071.67 ± 553.64
		486.64		357.95		1323.67	
		734.77		540.47		1998.59	
		947.08		696.63		2576.06	
C18	0.1	81.75	87.20 ± 33.60	20.44	21.81 ± 8.40	3.10	3.31 ± 1.27
		87.20		21.80		3.31	
		130.80		32.70		4.96	
		49.05		12.26		1.86	
	0.01	386.42	233.42 ± 176.40	13.41	8.10 ± 6.12	13.41	8.10 ± 6.12
		131.42		4.56		4.56	
		37.27		1.29		1.29	
		378.57		13.14		13.14	
	0.001	52.96	65.18 ± 17.53	2.71	3.33 ± 0.90	2.71	3.33 ± 0.90
		50.24		2.57		2.57	
		69.26		3.54		3.54	
		88.27		4.51		4.51	
	0.0001	348.28	167.14 ± 121.70	27.95	13.41 ± 9.77	19.59	9.40 ± 6.84
		120.76		9.69		6.79	
		113.76		9.13		6.40	
		85.76		6.88		4.82	

C20	0.1	279.79<br>539.10<br>757.47<br>59.47	408.96 ± 304.00	32.60<br>62.81<br>88.25<br>6.93	47.64 ± 35.41	32.60<br>62.81<br>88.25<br>6.93	47.64 ± 35.41
	0.01	82.89<br>54.79<br>15.45	51.05 ± 33.87	5.84<br>3.86<br>1.09	3.60 ± 2.39	5.84<br>3.86<br>1.09	3.60 ± 2.39
	0.001	71.49<br>58.00<br>724.38<br>799.93	413.45 ± 403.86	4.82<br>3.91<br>48.86<br>53.95	27.89 ± 27.24	4.82<br>3.91<br>48.86<br>53.95	27.89 ± 27.24
	0.0001	135.62<br>64.07<br>135.62<br>400.21	183.88 ± 148.11	12.43<br>5.87<br>12.43<br>36.67	16.85 ± 13.57	18.32<br>5.41<br>8.65<br>18.32	12.67 ± 6.65
C22	0.1	51.29<br>48.44<br>34.19<br>165.26	74.79 ± 60.77	3.82<br>3.60<br>2.54<br>12.30	5.56 ± 4.52	2.55<br>2.41<br>1.70<br>8.23	3.72 ± 3.02
	0.01	30.52<br>26.05<br>24.56<br>55.82	34.24 ± 14.61	2.79<br>2.38<br>2.25<br>5.11	3.13 ± 1.34	3.12<br>2.66<br>2.51<br>5.70	3.50 ± 1.49
	0.001	28.74<br>14.00<br>27.26<br>28.74	24.69 ± 7.16	2.65<br>1.29<br>2.52<br>2.65	2.28 ± 0.66	2.85<br>1.39<br>2.70<br>2.85	2.44 ± 0.71
	0.0001	38.63<br>47.61<br>26.05<br>45.81	39.52 ± 9.78	2.40<br>2.95<br>1.62<br>2.84	2.45 ± 0.61	3.27<br>4.03<br>2.21<br>3.88	3.35 ± 0.83
C24	0.1	167.75<br>55.28<br>101.03<br>165.84	122.48 ± 54.48	106.24<br>35.01<br>63.99<br>105.03	77.57 ± 34.51	158.80<br>52.33<br>95.64<br>157.00	115.94 ± 51.58
	0.01	47.38<br>42.39<br>52.36<br>42.39	46.13 ± 4.77	11.49<br>10.28<br>12.69<br>10.28	11.18 ± 1.16	10.29<br>9.20<br>11.37<br>9.20	10.01 ± 1.04
	0.001	21.33<br>26.07<br>24.49<br>41.87	28.44 ± 9.17	2.93<br>3.58<br>3.36<br>5.75	3.90 ± 1.26	2.73<br>3.34<br>3.14<br>5.36	3.64 ± 1.17
	0.0001	24.95<br>37.05<br>21.93<br>44.61	32.13 ± 10.58	1.12<br>1.67<br>0.99<br>2.01	1.45 ± 0.48	2.58<br>3.84<br>2.27<br>4.62	3.33 ± 1.09
C26	0.1	69.15<br>41.49<br>96.81<br>132.38	84.96 ± 38.85	7.10<br>4.26<br>9.95<br>13.60	8.73 ± 3.99	7.10<br>4.26<br>9.95<br>13.60	8.73 ± 3.99
	0.01	45.63<br>34.80<br>25.52<br>31.71	34.42 ± 8.41	3.88<br>2.96<br>2.17<br>2.69	2.93 ± 0.71	3.88<br>2.96<br>2.17<br>2.69	2.93 ± 0.71
	0.001	29.20<br>48.04<br>12.25<br>17.90	26.85 ± 15.79	2.41<br>3.96<br>1.01<br>1.48	2.21 ± 1.30	2.41<br>3.96<br>1.01<br>1.48	2.21 ± 1.30
	0.0001	20.96<br>47.44<br>47.44<br>29.79	36.41 ± 13.24	1.18<br>2.68<br>2.68<br>1.68	2.06 ± 0.75	1.31<br>2.98<br>2.98<br>1.87	2.28 ± 0.83
C28	0.1	67.79<br>59.14<br>59.14<br>24.52	52.65 ± 19.19	8.62<br>7.52<br>7.52<br>3.12	6.70 ± 2.44	8.62<br>7.52<br>7.52<br>3.12	6.70 ± 2.44
	0.01	38.67<br>41.53<br>98.82<br>64.45	60.87 ± 27.81	5.85<br>6.28<br>14.94<br>9.74	9.20 ± 4.20	5.85<br>6.28<br>14.94<br>9.74	9.20 ± 4.20
	0.001	49.30<br>57.04<br>43.50	218.97 ± 6.79	7.50<br>8.67<br>6.61	7.59 ± 1.03	7.50<br>8.67<br>6.61	7.59 ± 1.03
	0.0001	72.45<br>68.12<br>104.89<br>370.89	154.09 ± 145.46	9.14<br>8.59<br>13.23<br>46.77	19.43 ± 18.34	10.36<br>9.74<br>15.00<br>5.30	10.10 ± 3.97
C30	0.1	179.40<br>69.42<br>203.02<br>195.16	161.76 ± 62.33	49.44<br>19.13<br>55.94<br>53.78	44.57 ± 17.18	49.44<br>19.13<br>55.94<br>53.78	44.57 ± 17.18
	0.01	167.69<br>220.45<br>141.31<br>65.94	148.85 ± 64.32	12.20<br>16.03<br>10.28<br>4.80	10.83 ± 4.68	12.20<br>16.03<br>10.28<br>4.80	10.83 ± 4.68
	0.001	103.52<br>57.81<br>208.39<br>63.19	108.23 ± 69.82	7.02<br>3.92<br>14.14<br>4.29	7.34 ± 4.74	7.02<br>3.92<br>14.14<br>4.29	7.34 ± 4.74
	0.0001	178.49<br>227.54<br>123.99<br>110.36	160.10 ± 53.74	8.92<br>11.37<br>6.19<br>5.51	8.00 ± 2.69	8.03<br>10.23<br>5.58<br>4.96	7.20 ± 2.42

BO	0.1	26.38<br>26.38<br>22.47	27.36 ± 4.92	3.04<br>3.04<br>2.59<br>3.95	3.16 ± 0.57	3.04<br>3.04<br>2.59<br>3.95	3.16 ± 0.57
	0.01	36.25<br>50.75<br>63.18<br>21.75	42.98 ± 17.93	4.60<br>6.44<br>8.02<br>2.76	5.46 ± 2.28	4.60<br>6.44<br>8.02<br>2.76	5.46 ± 2.28
	0.001	41.17<br>72.40<br>46.85<br>55.37	53.95 ± 13.62	3.55<br>6.25<br>4.04<br>4.78	4.66 ± 1.17	3.55<br>6.25<br>4.04<br>4.78	4.66 ± 1.17
	0.0001	31.50<br>43.17<br>29.17<br>52.50	39.09 ± 10.84	2.83<br>3.88<br>2.62<br>4.71	3.51 ± 0.97	2.97<br>4.07<br>2.75<br>4.95	3.69 ± 1.02
BS	0.1	31.29<br>67.05<br>37.25<br>25.33	40.23 ± 18.53	2.31<br>4.95<br>2.75<br>1.87	2.97 ± 1.37	2.31<br>4.95<br>2.75<br>1.87	2.97 ± 1.37
	0.01	23.29<br>82.02<br>53.66<br>57.71	54.17 ± 24.10	2.46<br>8.68<br>5.68<br>6.11	5.73 ± 2.55	2.46<br>8.68<br>5.68<br>6.11	5.73 ± 2.55
	0.001	68.75<br>110.26<br>71.35<br>45.40	73.94 ± 26.88	5.08<br>8.14<br>5.27<br>3.35	5.46 ± 1.98	5.08<br>8.14<br>5.27<br>3.35	5.46 ± 1.98
	0.0001	48.71<br>78.81<br>73.50<br>43.39	61.10 ± 17.65	3.57<br>5.78<br>5.39<br>3.18	4.48 ± 1.29	4.95<br>8.01<br>7.47<br>4.41	6.21 ± 1.79
GO	0.1	63.38<br>162.41<br>79.23<br>75.26	95.07 ± 45.40	4.52<br>11.58<br>5.65<br>5.37	6.78 ± 3.24	4.52<br>11.58<br>5.65<br>5.37	6.78 ± 3.24
	0.01	48.85<br>40.70<br>48.85	46.13 ± 4.70	4.40<br>3.67<br>4.40<br>59.83	18.08 ± 27.83	4.40<br>3.67<br>4.40	4.16 ± 0.42
	0.001	61.72<br>65.58<br>115.73<br>3.86	61.72 ± 45.75	4.62<br>4.91<br>8.66	6.06 ± 2.25	4.62<br>4.91<br>8.66	6.06 ± 2.25
	0.0001	110.82<br>76.43<br>57.32<br>129.93	93.63 ± 32.80	11.46<br>7.91<br>5.93<br>13.44	9.68 ± 3.39	7.36<br>5.07<br>3.81<br>8.63	6.22 ± 2.18
S	0.1	55.77<br>57.46<br>77.74<br>148.72	84.92 ± 43.69	22.81<br>23.50<br>31.79<br>60.82	34.73 ± 17.86	22.81<br>23.50<br>31.79<br>60.82	34.73 ± 17.86
	0.01	49.07<br>22.08<br>56.43<br>31.89	39.87 ± 15.69	8.27<br>3.72<br>9.52<br>5.38	6.72 ± 2.65	8.27<br>3.72<br>9.52<br>5.38	6.72 ± 2.65
	0.001	61.97<br>46.48<br>74.88<br>136.85	80.04 ± 39.61	11.90<br>8.93<br>14.38<br>26.28	15.37 ± 7.61	11.90<br>8.93<br>14.38<br>26.28	15.37 ± 7.61
	0.0001	51.02<br>59.52<br>87.16	65.90 ± 18.90	10.64<br>12.41<br>18.17<br>42.55	20.94 ± 14.76	12.28<br>14.32<br>20.97<br>49.11	24.17 ± 17.03
TA	0.1	105.49<br>95.54<br>131.36	110.80 ± 18.49	18.03<br>16.33<br>22.45	18.93 ± 3.16	18.03<br>16.33<br>22.45	18.93 ± 3.16
	0.01	201.44<br>56.65<br>113.31<br>73.44	111.21 ± 64.67	25.45<br>7.16<br>14.32<br>9.28	14.05 ± 8.17	25.45<br>7.16<br>14.32<br>9.28	14.05 ± 8.17
	0.001	115.67<br>103.87<br>155.80<br>139.28	128.66 ± 23.33	13.78<br>12.37<br>18.56<br>16.59	15.33 ± 2.78	13.78<br>12.37<br>18.56<br>16.59	15.33 ± 2.78
	0.0001	23.88<br>81.60<br>83.59<br>103.50	73.14 ± 34.30	2.13<br>7.28<br>7.45<br>9.23	6.52 ± 3.06	2.62<br>8.97<br>9.19<br>11.37	8.04 ± 3.77

CC – cuticular compound; SD – standard deviation; SAB – *C. coronatus* colonies cultivated on Sabouraud agar medium; SAB-GM – *C. coronatus* colonies cultivated on Sabouraud agar medium with the addition of homogenized *G. mellonella* larvae; C10-C30 – fatty alcohols; BO – butyl oleate; BS – butyl stearate; GO – glycerol oleate; S – squalene; TA – tocopherol acetate
