# Supplementary material for: Insect cuticular compounds affect Conidiobolus coronatus (Entomopthorales) sporulation and the activity of enzymes involved in fungal infection
Source: Sci Rep. 2022 Aug 10;12:13641. doi: 10.1038/s41598-022-17960-z (PMC9365854; doi:10.1038/s41598-022-17960-z)
Supplement: Supplementary file 6 — Supplementary Information 6. [file 41598_2022_17960_MOESM6_ESM.pdf]

Supplementary Table 4. Chitobiosidase activity in *C. coronatus* conidia

CC		Total activity		Activity per protein content in one conidium		Activity in one conidium	
		Value (pM/min/ng)	Average value (pM/min/ng ± SD)	Value (pM/min/ng)	Average value (pM/min/ng ± SD)	Value pM/min/conidium)	Average value (pM/conidium ± SD)
SAB		28.84	47.36 ± 35.96	3.10	5.08 ± 3.86	3.26	5.36 ± 4.07
		32.45		3.48		3.67	
		24.07		2.58		2.72	
		38.49		4.13		4.35	
		81.21		8.72		9.19	
		13.61		1.46		1.54	
		112.85		12.11		12.77	
SAB-GM		23.38	28.51 ± 15.91	3.60	4.39 ± 2.45	3.67	4.47 ± 2.50
		32.48		5.00		5.09	
		25.85		3.98		4.05	
		53.50		8.24		8.39	
		8.99		1.38		1.41	
		12.47		1.92		1.96	
		42.93		6.62		6.73	
C10	0.1	lack of growth					
	0.01	16.14	31.67 ± 19.65	2.90	5.68 ± 3.53	2.77	5.44 ± 3.38
		31.96		5.73		5.49	
		59.30		10.64		10.19	
		19.26		3.46		3.31	
	0.001	47.55	28.01 ± 14.13	4.67	2.75 ± 1.39	4.67	2.75 ± 1.39
		28.97		2.85		2.85	
		15.96		1.57		1.57	
		19.57		1.92		1.92	
	0.0001	16.24	23.49 ± 19.84	1.39	2.01 ± 1.70	1.95	2.82 ± 2.38
		7.85		0.67		0.94	
		17.31		1.49		2.08	
		52.56		4.51		6.31	
C12	0.1	28.00	25.48 ± 6.91	5.65	5.15 ± 1.39	5.65	5.15 ± 1.39
		15.93		3.22		3.22	
		32.23		6.51		6.51	
		25.76		5.20		5.20	
	0.01	10.81	7.28 ± 2.84	2.37	1.59 ± 0.62	2.11	1.42 ± 0.55
		8.23		1.80		1.61	
		4.42		0.97		0.86	
		5.65		1.24		1.10	
	0.001	1.78	4.45 ± 2.31	0.21	0.53 ± 0.28	0.21	0.53 ± 0.28
		7.35		0.88		0.88	
		3.90		0.47		0.47	
		4.79		0.57		0.57	
	0.0001	11.37	7.50 ± 4.02	0.98	0.65 ± 0.35	0.77	0.51 ± 0.27
		10.58		0.91		0.71	
		4.10		0.35		0.28	
		3.97		0.34		0.27	
C14	0.1	4.27	5.90 ± 3.58	0.32	0.44 ± 0.27	0.32	0.44 ± 0.27
		10.82		0.81		0.81	
		2.50		0.19		0.19	
		6.03		0.45		0.45	
	0.01	3.53	5.25 ± 1.76	0.21	0.32 ± 0.11	0.22	0.33 ± 0.11
		4.05		0.24		0.26	
		7.27		0.44		0.46	
		6.13		0.37		0.39	
	0.001	6.15	5.34 ± 1.89	0.61	0.53 ± 0.19	0.61	0.53 ± 0.19
		4.66		0.46		0.46	
		3.07		0.30		0.30	
		7.46		0.74		0.74	
	0.0001	5.11	7.27 ± 2.00	0.25	0.35 ± 0.10	0.50	0.71 ± 0.19
		6.08		0.29		0.59	
		8.54		0.41		0.83	
		9.36		0.45		0.91	
C16	0.1	358.02	259.51 ± 151.84	216.24	156.74 ± 91.71	216.24	156.74 ± 91.71
		38.51		23.26		23.26	
		360.23		217.58		217.58	
		281.30		169.90		169.90	
	0.01	381.34	266.73 ± 144.93	212.20	148.42 ± 80.65	212.20	148.42 ± 80.65
		199.28		110.89		110.89	
		94.46		52.56		52.56	
		391.84		218.04		218.04	
	0.001	358.59	341.12 ± 45.88	358.84	341.36 ± 45.91	364.98	347.20 ± 46.70
		272.56		272.75		277.42	
		368.21		368.47		374.78	
		365.12		365.38		371.63	
	0.0001	278.49	272.67 ± 12.89	204.85	200.57 ± 9.48	757.50	741.67 ± 35.05
		253.35		186.35		689.12	
		279.31		205.45		759.73	
		279.52		205.61		760.31	
C18	0.1	35.62	37.79 ± 10.25	8.91	9.45 ± 2.56	1.35	1.43 ± 0.39
		27.36		6.84		1.04	
		51.90		12.98		1.97	
		36.27		9.07		1.38	
	0.01	27.51	17.66 ± 7.27	0.95	0.61 ± 0.25	0.95	0.61 ± 0.25
		15.32		0.53		0.53	
		10.16		0.35		0.35	
		17.66		0.61		0.61	
	0.001	9.09	9.50 ± 0.87	0.46	0.48 ± 0.04	0.46	0.48 ± 0.04
		9.09		0.46		0.46	
		10.61		0.54		0.54	
				0.47		0.47	
	0.0001	11.44	11.51 ± 3.00	0.92	0.92 ± 0.24	0.64	0.65 ± 0.17
		9.76		0.78		0.55	
		9.07		0.73		0.51	
		15.76		1.26		0.89	

**C20**	0.1	13.75<br>11.73<br>4.35	9.94 ± 4.95	1.60<br>1.37<br>0.51	1.16 ± 0.58	1.60<br>1.37<br>0.51	1.16 ± 0.58
	0.01	11.87<br>27.43<br>44.56<br>7.95	22.95 ± 16.68	0.84<br>1.93<br>3.14<br>0.56	1.62 ± 1.18	0.84<br>1.93<br>3.14<br>0.56	1.62 ± 1.18
	0.001	6.13<br>6.77<br>5.70<br>2.26	5.21 ± 2.02	0.41<br>0.45<br>0.38<br>0.15	0.35 ± 0.14	0.41<br>0.45<br>0.38<br>0.15	0.35 ± 0.14
	0.0001	4.18<br>2.85<br>4.38<br>9.42	5.20 ± 2.89	0.38<br>0.26<br>0.40<br>0.86	0.48 ± 0.26	0.56<br>0.38<br>0.59<br>1.27	0.70 ± 0.39
**C22**	0.1	14.65<br>9.54<br>17.26<br>11.81	13.31 ± 3.36	1.09<br>0.71<br>1.28<br>0.88	0.99 ± 0.25	0.73<br>0.47<br>0.86<br>0.59	0.66 ± 0.17
	0.01	10.85<br>23.55<br>21.71<br>5.16	15.32 ± 8.79	0.99<br>2.15<br>1.99<br>0.47	1.40 ± 0.80	1.11<br>2.41<br>2.22<br>0.53	1.56 ± 0.90
	0.001	13.86<br>9.57<br>10.75<br>3.76	9.48 ± 4.22	1.28<br>0.88<br>0.99<br>0.35	0.88 ± 0.39	1.37<br>0.95<br>1.06<br>0.37	0.94 ± 0.42
	0.0001	20.90<br>21.33<br>6.23<br>14.25	15.68 ± 7.09	1.30<br>1.32<br>0.39<br>0.88	0.97 ± 0.44	1.77<br>1.81<br>0.53<br>1.21	1.33 ± 0.60
**C24**	0.1	12.30<br>19.98<br>13.37<br>19.60	16.31 ± 4.04	7.79<br>12.65<br>8.47<br>12.41	10.33 ± 2.56	11.65<br>18.91<br>12.66<br>18.55	15.44 ± 3.82
	0.01	12.72<br>11.53<br>12.52<br>10.00	11.69 ± 1.24	3.08<br>2.79<br>3.03<br>2.42	2.83 ± 0.30	2.76<br>2.50<br>2.72<br>2.17	2.54 ± 0.27
	0.001	13.09<br>11.08<br>13.41<br>16.62	13.55 ± 2.29	1.90<br>1.52<br>1.84<br>2.28	1.86 ± 0.31	1.68<br>1.42<br>1.72<br>2.13	1.73 ± 0.29
	0.0001	7.05<br>9.28<br>7.65<br>10.36	8.59 ± 1.51	0.32<br>0.42<br>0.34<br>0.47	0.39 ± 0.07	0.73<br>0.96<br>0.79<br>1.07	0.89 ± 0.16
**C26**	0.1	16.38<br>12.60<br>19.84<br>22.83	17.91 ± 4.42	1.68<br>1.29<br>2.04<br>2.35	1.84 ± 0.45	1.68<br>1.29<br>2.04<br>2.35	1.84 ± 0.45
	0.01	31.06<br>16.46<br>17.63<br>4.99	17.53 ± 10.67	2.64<br>1.40<br>1.50<br>0.42	1.49 ± 0.91	2.64<br>1.40<br>1.50<br>0.42	1.49 ± 0.91
	0.001	11.11<br>13.06<br>11.79<br>22.67	14.66 ± 5.40	0.92<br>1.08<br>0.97<br>1.87	1.21 ± 0.44	0.92<br>1.08<br>0.97<br>1.87	1.21 ± 0.44
	0.0001	5.36<br>24.80<br>18.29<br>12.13	15.15 ± 8.32	0.30<br>1.40<br>1.03<br>0.69	0.86 ± 0.47	0.34<br>1.56<br>1.15<br>0.76	0.95 ± 0.52
**C28**	0.1	9.89<br>16.32<br>12.76<br>17.70	14.17 ± 3.53	1.26<br>2.08<br>1.62<br>2.25	1.80 ± 0.45	1.26<br>2.08<br>1.62<br>2.25	1.80 ± 0.45
	0.01	15.18<br>11.19<br>7.30<br>33.10	16.69 ± 11.40	2.29<br>1.69<br>1.10<br>5.00	2.52 ± 1.72	2.29<br>1.69<br>1.10<br>5.00	2.52 ± 1.72
	0.001	17.33<br>15.95<br>12.17<br>7.40	13.21 ± 4.45	2.63<br>2.42<br>1.85<br>1.12	2.01 ± 0.68	2.63<br>2.42<br>1.85<br>1.12	2.01 ± 0.68
	0.0001	14.56<br>7.07<br>12.84<br>23.96	14.61 ± 7.01	1.84<br>0.89<br>1.62<br>3.02	1.84 ± 0.88	2.08<br>1.01<br>1.84<br>3.43	2.09 ± 1.00
**C30**	0.1	43.01<br>18.58<br>32.36<br>41.75	33.92 ± 11.28	11.85<br>5.12<br>8.92<br>11.50	9.35 ± 3.11	11.85<br>5.12<br>8.92<br>11.50	9.35 ± 3.11
	0.01	23.57<br>11.86<br>11.41<br>12.01	14.71 ± 5.91	1.71<br>0.86<br>0.83<br>0.87	1.07 ± 0.43	1.71<br>0.86<br>0.83<br>0.87	1.07 ± 0.43
	0.001	31.72<br>1.28<br>20.14	17.71 ± 15.36	2.15<br>0.09<br>1.37	1.20 ± 1.04	2.15<br>0.09<br>1.37	1.20 ± 1.04
	0.0001	20.85<br>15.64<br>7.71<br>15.53	14.93 ± 5.42	1.04<br>0.78<br>0.39<br>0.78	0.75 ± 0.27	0.94<br>0.70<br>0.35<br>0.70	0.67 ± 0.24

BO	0.1	6.81<br>12.34<br>10.63<br>8.84	9.66 ± 2.37	0.79<br>1.42<br>1.23<br>1.02	1.11 ± 0.27	0.79<br>1.42<br>1.23<br>1.02	1.11 ± 0.27
	0.01	8.46<br>8.63<br>26.29<br>6.73	12.53 ± 9.22	1.07<br>1.09<br>3.34<br>0.85	1.59 ± 1.17	1.07<br>1.09<br>3.34<br>0.85	1.59 ± 1.17
	0.001	9.56<br>18.05<br>8.88<br>14.43	12.73 ± 4.32	0.82<br>1.56<br>0.77<br>1.24	1.10 ± 0.37	0.82<br>1.56<br>0.77<br>1.24	1.10 ± 0.37
	0.0001	9.07<br>13.16<br>16.04<br>8.69	11.74 ± 3.51	0.81<br>1.18<br>1.44<br>0.78	1.05 ± 0.31	0.85<br>1.24<br>1.51<br>0.82	1.11 ± 0.33
BS	0.1	29.63<br>7.30<br>19.53<br>11.46	16.98 ± 9.84	2.19<br>0.54<br>1.44<br>0.85	1.25 ± 0.73	2.19<br>0.54<br>1.44<br>0.85	1.25 ± 0.73
	0.01	9.40<br>26.91<br>23.93<br>15.69	18.98 ± 7.96	0.99<br>2.85<br>2.53<br>1.66	2.01 ± 0.84	0.99<br>2.85<br>2.53<br>1.66	2.01 ± 0.84
	0.001	16.90<br>7.39<br>18.97<br>14.83	14.52 ± 5.05	1.25<br>0.55<br>1.40<br>1.09	1.07 ± 0.37	1.25<br>0.55<br>1.40<br>1.09	1.07 ± 0.37
	0.0001	10.76<br>19.73<br>24.67<br>11.68	16.71 ± 6.66	0.79<br>1.45<br>1.81<br>0.86	1.23 ± 0.49	1.09<br>2.00<br>2.51<br>1.19	1.70 ± 0.68
GO	0.1	6.08<br>12.07<br>22.49<br>11.29	12.98 ± 6.88	0.43<br>0.86<br>1.60<br>0.80	0.92 ± 0.49	0.43<br>0.86<br>1.60<br>0.80	0.92 ± 0.49
	0.01	11.76<br>4.62<br>7.54<br>15.81	9.93 ± 4.89	1.06<br>0.42<br>0.68<br>1.43	0.89 ± 0.44	1.06<br>0.42<br>0.68<br>1.43	0.89 ± 0.44
	0.001	25.75<br>18.83<br>16.83<br>11.14	18.14 ± 6.03	1.93<br>1.41<br>1.26<br>0.83	1.36 ± 0.45	1.93<br>1.41<br>1.26<br>0.83	1.36 ± 0.45
	0.0001	33.27<br>23.98<br>23.83<br>19.57	25.16 ± 5.78	3.44<br>2.48<br>2.47<br>2.02	2.60 ± 0.60	2.21<br>1.59<br>1.58<br>1.30	1.67 ± 0.38
S	0.1	11.55<br>7.51<br>9.12<br>28.18	14.09 ± 9.54	4.72<br>3.07<br>3.73<br>11.52	5.76 ± 3.90	4.72<br>3.07<br>3.73<br>11.52	5.76 ± 3.90
	0.01	7.48<br>15.40<br>6.99	9.96 ± 4.72	1.26<br>2.60<br>1.18<br>0.40	1.36 ± 0.91	1.26<br>0.40<br>2.60<br>1.18	1.36 ± 0.91
	0.001	13.43<br>8.90<br>9.62<br>21.86	13.45 ± 5.95	2.58<br>1.71<br>1.85<br>4.20	2.58 ± 1.14	2.58<br>1.71<br>1.85<br>4.20	2.58 ± 1.14
	0.0001	7.07<br>13.34<br>13.26<br>10.55	11.05 ± 2.95	1.47<br>2.78<br>2.76<br>2.20	2.30 ± 0.62	1.70<br>3.21<br>3.19<br>2.54	2.66 ± 0.71
TA	0.1	25.62<br>25.86<br>18.95<br>30.38	25.20 ± 4.70	4.38<br>4.42<br>3.24<br>5.19	4.31 ± 0.80	4.38<br>4.42<br>3.24<br>5.19	4.31 ± 0.80
	0.01	8.53<br>3.68<br>14.72<br>4.35	7.82 ± 5.07	1.08<br>0.46<br>1.86<br>0.55	0.99 ± 0.64	1.08<br>0.46<br>1.86<br>0.55	0.99 ± 0.64
	0.001	12.04<br>8.09<br>6.30<br>7.81	8.56 ± 2.45	1.43<br>0.96<br>0.75<br>0.93	1.02 ± 0.29	1.43<br>0.96<br>0.75<br>0.93	1.02 ± 0.29
	0.0001	14.67<br>6.98<br>5.39<br>30.06	9.01 ± 11.27	1.31<br>0.62<br>0.48<br>2.68	1.27 ± 1.01	1.61<br>0.77<br>0.59<br>3.30	1.57 ± 1.24

CC – cuticular compound; SD – standard deviation; SAB – *C. coronatus* colonies cultivated on Sabouraud agar medium; SAB-GM – *C. coronatus* colonies cultivated on Sabouraud agar medium with the addition of homogenized *G. mellonella* larvae; C10-C30 – fatty alcohols; BO – butyl oleate; BS – butyl stearate; GO – glycerol oleate; S – squalene; TA – tocopherol acetate
