# Supplementary material for: Insect cuticular compounds affect Conidiobolus coronatus (Entomopthorales) sporulation and the activity of enzymes involved in fungal infection
Source: Sci Rep. 2022 Aug 10;12:13641. doi: 10.1038/s41598-022-17960-z (PMC9365854; doi:10.1038/s41598-022-17960-z)
Supplement: Supplementary file 7 — Supplementary Information 7. [file 41598_2022_17960_MOESM7_ESM.pdf]

Supplementary Table 5. Lipase activity in *C. coronatus* conidia

CC		Total activity		Activity per protein content in one conidium		Activity in one conidium	
		Value (pM/min/ng)	Average value (pM/min/ng ± SD)	Value (pM/min/ng)	Average value (pM/min/ng ± SD)	Value (pM/min/conidium)	Average value (pM/conidium ± SD)
SAB		9.06	5.60 ± 3.03	0.97	0.60 ± 0.32	0.97	0.60 ± 0.32
		8.06		0.86		0.86	
		3.45		0.37		0.37	
		9.13		0.98		0.98	
		2.38		0.25		0.25	
		4.38		0.47		0.47	
		2.72		0.29		0.29	
SAB-GM		1.64	2.01 ± 1.26	0.25	0.31 ± 0.19	0.25	0.31 ± 0.19
		1.52		0.23		0.23	
		2.13		0.33		0.33	
		4.46		0.69		0.69	
		0.75		0.12		0.12	
		0.91		0.14		0.14	
		2.61		0.40		0.40	
C10	0.1	lack of growth					
	0.01	26.56	34.24 ± 17.56	4.77	6.14 ± 3.15	4.56	5.88 ± 3.02
		41.81		7.50		7.18	
		54.41		9.76		9.35	
		14.19		2.54		2.44	
	0.001	47.20	22.95 ± 21.38	4.64	2.25 ± 2.11	0.46	0.86 ± 0.52
		14.82		1.46		1.46	
		6.82		0.67		0.67	
	0.0001	13.39	9.68 ± 4.46	1.15	0.83 ± 0.38	1.61	1.16 ± 0.54
		3.47		0.30		0.42	
		9.46		0.81		1.14	
		12.40		1.06		1.49	
C12	0.1	5.02	7.61 ± 3.68	1.01	1.54 ± 0.74	1.01	1.54 ± 0.74
		13.06		2.64		2.64	
		6.50		1.31		1.31	
		5.85		1.18		1.18	
	0.01	lack of activity					
	0.001	lack of activity					
	0.0001	lack of activity					
C14	0.1	7.76	23.72 ± 18.03	0.58	1.77 ± 1.35	0.58	1.21 ± 1.08
		43.28		3.23		2.70	
		20.12		1.50		1.26	
						0.28	
	0.01	33.30	15.95 ± 12.13	2.02	0.96 ± 0.73	2.11	1.01 ± 0.77
		6.57		0.40		0.42	
		15.19		0.92		0.96	
		8.74		0.53		0.55	
	0.001	6.94	6.49 ± 2.26	0.69	0.63 ± 0.22	0.69	0.63 ± 0.22
		3.61		0.36		0.36	
		5.98		0.59		0.59	
		9.06		0.90		0.90	
	0.0001	10.68	13.30 ± 2.86	0.51	0.64 ± 0.14	1.04	1.30 ± 0.28
		12.14		0.58		1.18	
		17.34		0.84		1.69	
		13.03		0.63		1.27	
C16	0.1	1.87	1.98 ± 0.58	1.13	1.20 ± 0.35	1.13	1.20 ± 0.35
		2.61		1.58		1.58	
		1.47		0.89		0.89	
	0.01	2.36	1.86 ± 1.08	1.31	1.03 ± 0.60	1.31	1.03 ± 0.60
		0.97		0.54		0.54	
		0.95		0.53		0.53	
		3.14		1.75		1.75	
	0.001	4.38	4.60 ± 0.57	4.37	4.61 ± 0.59	4.46	4.68 ± 0.60
		5.47		5.47		5.56	
		4.15		4.15		4.23	
		4.41		4.41		4.49	
	0.0001	3.37	3.33 ± 0.18	2.48	2.45 ± 0.13	9.17	9.07 ± 0.48
		3.21		2.36		8.72	
		3.57		2.63		9.71	
		3.19		2.34		8.67	
C18	0.1	1.57	4.21 ± 2.61	0.39	1.05 ± 0.65	0.39	1.05 ± 0.65
		7.35		1.84		1.84	
		2.64		0.66		0.66	
		5.28		1.32		1.32	
	0.01	6.44	6.52 ± 2.99	0.22	0.23 ± 0.10	0.22	0.23 ± 0.10
		9.55		0.33		0.33	
		3.57		0.12		0.12	
	0.001	3.43	7.13 ± 4.79	0.17	0.36 ± 0.24	0.17	0.36 ± 0.24
		12.54		0.64		0.64	
		5.41		0.28		0.28	
	0.0001	2.14	1.19 ± 0.97	0.17	0.10 ± 0.08	0.12	0.07 ± 0.05
		1.24		0.10		0.07	
		0.20		0.02		0.01	

C20	0.1	8.32<br>12.16<br>21.56	14.01 ± 6.81	0.97<br>1.42<br>0.82	1.07 ± 0.31	0.97<br>1.42<br>0.82	1.07 ± 0.31
	0.01	9.16<br>25.94<br>61.25	32.12 ± 26.59	0.64<br>1.83<br>4.32	2.26 ± 1.87	0.64<br>1.83<br>4.32	2.26 ± 1.87
	0.001	0.43<br>2.85<br>1.24<br>4.79	2.33 ± 1.93	0.03<br>0.19<br>0.08<br>0.32	0.16 ± 0.13	0.03<br>0.19<br>0.08<br>0.32	0.16 ± 0.13
	0.0001	1.78<br>1.43<br>3.73<br>4.48	2.85 ± 1.48	0.16<br>0.13<br>0.34<br>0.41	0.26 ± 0.14	0.24<br>0.19<br>0.50<br>0.60	0.38 ± 0.20
C22	0.1	10.43<br>15.98<br>9.55<br>32.62	17.14 ± 10.70	0.78<br>1.19<br>0.71<br>2.43	1.27 ± 0.80	0.52<br>0.79<br>0.47<br>1.62	0.85 ± 0.53
	0.01	4.04<br>2.17<br>2.23<br>2.38	2.70 ± 0.89	0.37<br>0.20<br>0.20<br>0.22	0.25 ± 0.08	0.41<br>0.22<br>0.23<br>0.24	0.28 ± 0.09
	0.001	4.50<br>1.33<br>4.77<br>1.49	3.02 ± 1.87	0.41<br>0.12<br>0.44<br>0.14	0.28 ± 0.17	0.44<br>0.13<br>0.47<br>0.15	0.30 ± 0.18
	0.0001	2.52<br>3.38<br>4.08<br>2.84	3.20 ± 0.68	0.16<br>0.21<br>0.25<br>0.18	0.20 ± 0.04	0.21<br>0.28<br>0.34<br>0.24	0.27 ± 0.06
C24	0.1	3.20<br>4.00<br>3.16<br>3.36	3.43 ± 0.40	2.03<br>2.54<br>2.00<br>2.13	2.17 ± 0.25	3.03<br>3.79<br>2.99<br>3.18	3.25 ± 0.37
	0.01	2.99<br>5.73<br>9.03<br>2.40	5.04 ± 3.03	0.72<br>1.39<br>2.19<br>0.58	1.22 ± 0.73	0.65<br>1.24<br>1.96<br>0.52	1.09 ± 0.66
	0.001	4.86<br>1.26<br>1.31	2.47 ± 2.06	0.67<br>0.17<br>0.18	0.34 ± 0.28	1.54<br>0.62<br>0.16<br>0.17	0.62 ± 0.65
	0.0001	7.53<br>6.19<br>2.67<br>6.24	5.66 ± 2.08	0.34<br>0.28<br>0.12<br>0.28	0.25 ± 0.09	0.78<br>0.64<br>0.28<br>0.65	0.59 ± 0.22
C26	0.1	1.53<br>5.07<br>2.01<br>3.15	2.94 ± 1.57	0.16<br>0.52<br>0.21<br>0.32	0.30 ± 0.16	0.16<br>0.52<br>0.21<br>0.32	0.30 ± 0.16
	0.01	6.57<br>1.55<br>0.93	3.02 ± 3.09	0.56<br>0.13<br>0.08	0.26 ± 0.26	0.56<br>0.13<br>0.08	0.26 ± 0.26
	0.001	0.88<br>0.73<br>1.04	0.88 ± 0.15	0.07<br>0.06<br>0.08	0.07 ± 0.01	0.07<br>0.41<br>0.06	0.07 ± 0.01
	0.0001	0.24<br>0.69<br>1.28<br>0.81	0.75 ± 0.43	0.01<br>0.04<br>0.07<br>0.05	0.04 ± 0.02	0.01<br>0.04<br>0.08<br>0.05	0.05 ± 0.03
C28	0.1	1.74<br>2.21<br>2.21<br>1.71	1.97 ± 0.28	0.22<br>0.28<br>0.28<br>0.22	0.25 ± 0.04	0.22<br>0.28<br>0.28<br>0.22	0.25 ± 0.04
	0.01	4.65<br>3.64<br>2.76<br>5.35	4.10 ± 1.14	0.70<br>0.55<br>0.42<br>0.81	0.62 ± 0.17	0.70<br>0.55<br>0.42<br>0.81	0.62 ± 0.17
	0.001	11.48<br>9.07<br>2.26<br>3.75	6.64 ± 4.35	1.74<br>1.38<br>0.34<br>0.57	1.01 ± 0.66	1.74<br>1.38<br>0.34<br>0.57	1.01 ± 0.66
	0.0001	4.33<br>7.05<br>13.40<br>1.99	6.69 ± 4.92	0.55<br>0.89<br>1.69<br>0.25	0.84 ± 0.62	0.62<br>1.01<br>1.92<br>0.28	0.96 ± 0.70
C30	0.1	5.35<br>4.28<br>5.11<br>7.32	5.52 ± 1.29	1.47<br>1.18<br>1.41<br>2.02	1.52 ± 0.35	1.47<br>1.18<br>1.41<br>2.02	1.52 ± 0.35
	0.01	12.01<br>29.36<br>7.39<br>7.04	13.95 ± 10.52	0.87<br>2.13<br>0.54<br>0.51	1.01 ± 0.76	0.87<br>2.13<br>0.54<br>0.51	1.01 ± 0.76
	0.001	17.10<br>11.45<br>6.80<br>11.12	11.62 ± 4.22	1.16<br>0.78<br>0.46<br>0.75	0.79 ± 0.29	1.16<br>0.78<br>0.46<br>0.75	0.79 ± 0.29
	0.0001	14.10<br>12.28<br>14.61<br>9.39	12.59 ± 2.36	0.70<br>0.61<br>0.73<br>0.47	0.63 ± 0.12	0.63<br>0.55<br>0.66<br>0.42	0.57 ± 0.11

BO	0.1	15.09<br>15.61<br>15.09	15.26 ± 0.30	1.74<br>1.80<br>1.74	1.76 ± 0.03	1.74<br>1.80<br>1.74	1.76 ± 0.03
	0.01	11.57<br>11.64<br>17.87<br>28.45	17.38 ± 7.95	1.47<br>1.48<br>2.27<br>3.61	2.21 ± 1.01	1.47<br>1.48<br>2.27<br>3.61	2.21 ± 1.01
	0.001	6.13<br>17.00<br>18.87<br>51.83	23.46 ± 19.73	0.53<br>1.47<br>1.63<br>4.47	2.00 ± 1.70	0.53<br>1.47<br>1.63<br>4.47	2.00 ± 1.70
	0.0001	2.27<br>13.83<br>20.17<br>44.52	20.20 ± 17.83	0.20<br>1.24<br>1.81<br>4.00	1.81 ± 1.60	0.20<br>1.30<br>1.90<br>0.42	0.96 ± 0.79
BS	0.1	7.17<br>7.07<br>9.53	7.92 ± 1.39	0.53<br>0.52<br>0.70	0.58 ± 0.10	0.53<br>0.52<br>0.70	0.58 ± 0.10
	0.01	11.50<br>5.62<br>6.64	7.92 ± 3.14	1.22<br>0.59<br>0.70	0.84 ± 0.33	1.22<br>0.59<br>0.70	0.84 ± 0.33
	0.001	3.17<br>3.99<br>7.31<br>21.23	8.92 ± 8.39	0.23<br>0.29<br>0.54<br>1.57	0.66 ± 0.62	0.23<br>0.29<br>0.54<br>1.57	0.66 ± 0.62
	0.0001	14.80<br>15.48<br>26.86<br>21.39	19.63 ± 5.65	1.09<br>1.14<br>1.97<br>1.57	1.44 ± 0.41	1.50<br>1.57<br>2.73<br>2.17	1.99 ± 0.57
GO	0.1	1.86<br>1.86<br>1.30<br>2.71	1.99 ± 0.51	0.13<br>0.13<br>0.11<br>0.19	0.14 ± 0.04	0.13<br>0.13<br>0.11<br>0.19	0.14 ± 0.04
	0.01	2.00<br>2.28<br>5.22<br>2.91	3.10 ± 1.46	0.18<br>0.21<br>0.47<br>0.26	0.28 ± 0.13	0.18<br>0.21<br>0.47<br>0.26	0.28 ± 0.13
	0.001	10.59<br>2.48<br>2.96	5.34 ± 4.55	0.79<br>0.18<br>0.22	0.40 ± 0.34	0.79<br>0.18<br>0.22	0.40 ± 0.34
	0.0001	4.66<br>8.80<br>1.83<br>6.16	5.36 ± 2.91	0.48<br>0.91<br>0.19<br>0.64	0.55 ± 0.30	0.31<br>0.58<br>0.12<br>0.41	0.36 ± 0.19
S	0.1	4.72<br>7.21<br>3.52<br>3.14	4.65 ± 1.84	1.93<br>2.95<br>1.44<br>1.28	1.90 ± 0.75	1.93<br>2.95<br>1.44<br>1.28	1.90 ± 0.75
	0.01	2.29<br>4.48<br>3.70<br>6.63	4.28 ± 1.81	0.38<br>0.76<br>0.62<br>1.12	0.72 ± 0.31	0.38<br>0.76<br>0.62<br>1.12	0.72 ± 0.31
	0.001	9.13<br>1.50<br>3.09<br>4.40	4.53 ± 3.29	1.75<br>0.29<br>0.59<br>0.84	0.87 ± 0.63	1.75<br>0.29<br>0.59<br>0.84	0.87 ± 0.63
	0.0001	3.16<br>0.86<br>1.76<br>1.10	1.72 ± 1.03	0.66<br>0.18<br>0.37<br>0.23	0.36 ± 0.22	0.70<br>0.21<br>0.42<br>0.27	0.41 ± 0.25
TA	0.1	0.91<br>1.71<br>1.44	1.36 ± 0.41	0.15<br>0.29<br>0.25	0.23 ± 0.07	0.15<br>0.29<br>0.25	0.23 ± 0.07
	0.01	2.88<br>0.36<br>0.59<br>0.90	1.18 ± 1.15	0.36<br>0.04<br>0.07<br>0.11	0.07 ± 0.03	0.36<br>0.04<br>0.07<br>0.11	0.07 ± 0.03
	0.001	2.03<br>1.32<br>1.15<br>0.59	1.28 ± 0.59	0.24<br>0.16<br>0.14<br>0.07	0.15 ± 0.07	0.24<br>0.16<br>0.14<br>0.07	0.15 ± 0.07
	0.0001	0.83<br>0.48<br>1.92<br>3.25	1.62 ± 1.25	0.07<br>0.04<br>0.17<br>0.29	0.14 ± 0.11	0.09<br>0.05<br>0.21<br>0.36	0.18 ± 0.14

CC – cuticular compound; SD – standard deviation; SAB – *C. coronatus* colonies cultivated on Sabouraud agar medium; SAB-GM – *C. coronatus* colonies cultivated on Sabouraud agar medium with the addition of homogenized *G. mellonella* larvae; C10-C30 – fatty alcohols; BO – butyl oleate; BS – butyl stearate; GO – glycerol oleate; S – squalene; TA – tocopherol acetate
